# Oxytocin-pathway polygenic scores for severe mental disorders and metabolic phenotypes in the UK Biobank

**DOI:** 10.1038/s41398-021-01725-9

**Published:** 2021-11-25

**Authors:** Adriano Winterton, Francesco Bettella, Ann-Marie G. de Lange, Marit Haram, Nils Eiel Steen, Lars T. Westlye, Ole A. Andreassen, Daniel S. Quintana

**Affiliations:** 1 Norwegian Centre for Mental Disorders Research (NORMENT), University of Oslo and Oslo University Hospital, Oslo, Norway; 2 Department of Psychology, University of Oslo, Oslo, Norway; 3 Department of Psychiatry, University of Oxford, Oxford, UK; 4 KG Jebsen Centre for Neurodevelopmental Disorders, University of Oslo, Oslo, Norway; 5 NevSom, Department of Rare Disorders, Oslo University Hospital, Oslo, Norway

**Keywords:** Genomics, Psychiatric disorders

## Abstract

Oxytocin is a neuromodulator and hormone that is typically associated with social cognition and behavior. In light of its purported effects on social cognition and behavior, research has investigated its potential as a treatment for psychiatric illnesses characterized by social dysfunction, such as schizophrenia and bipolar disorder. While the results of these trials have been mixed, more recent evidence suggests that the oxytocin system is also linked with cardiometabolic conditions for which individuals with severe mental disorders are at a higher risk for developing. To investigate whether the oxytocin system has a pleiotropic effect on the etiology of severe mental illness and cardiometabolic conditions, we explored oxytocin’s role in the shared genetic liability of schizophrenia, bipolar disorder, type-2 diabetes, and several phenotypes linked with cardiovascular disease and type 2 diabetes risk using a polygenic pathway-specific approach. Analysis of a large sample with about 480,000 individuals (UK Biobank) revealed statistically significant associations across the range of phenotypes analyzed. By comparing these effects to those of polygenic scores calculated from 100 random gene sets, we also demonstrated the specificity of many of these significant results. Altogether, our results suggest that the shared effect of oxytocin-system dysfunction could help partially explain the co-occurrence of social and cardiometabolic dysfunction in severe mental illnesses.

## Introduction

Schizophrenia (SCZ) and bipolar disorders (BD) are associated with reduced life expectancies [1, 2], partly caused by an increased risk for cardiovascular disease (CVD) [3]. One-third of patients with psychotic disorders suffer from metabolic syndrome (MetS) [4], a collection of co-occurring metabolic risk factors [5] (i.e., glucose intolerance, insulin resistance, visceral adiposity, dyslipidemia, and hypertension) for the development of CVD and type-2 diabetes mellitus (T2D) [5]. Studies have reported a two-time higher prevalence of MetS in people with bipolar disorder or schizophrenia [6], and this increased prevalence is comparable between males and females [7].

In addition to lifestyle habits such as smoking, poor diet, and a lack of exercise [8], some antipsychotic medications account for a portion of the increased prevalence of MetS risk factors in these patients [9]. However, evidence of MetS risk factors in untreated individuals with first-episode psychosis [10], in antipsychotic-naive patients [11], and in healthy first-degree relatives [12] suggests that the risk factors are, in part, independent from antipsychotic treatments and lifestyle factors, which point to other influences. While genetic studies support the presence of common causes predisposing individuals to both MetS risk factors, and psychotic disorders [13], the mechanisms underpinning the shared risk for psychotic disorders and MetS remain unclear. An additional unexplained piece of the puzzle is the prevalence of loneliness and social isolation among patients with psychotic disorders—the annual rate of loneliness is approximately 2.3 times higher in patients with schizophrenia and bipolar disorder than in the general population [14], and research has demonstrated a link between loneliness, MetS risk factors and cardiovascular morbidity [15]. However, little is known about the mechanisms underlying this association.

Emerging evidence suggests that oxytocin-system dysfunction might play a pleiotropic role in the etiology of psychotic disorders and MetS risk factors [16–18]. Oxytocin is a versatile and multifunctional hormone and neurotransmitter associated with a variety of social behaviors and employed in several therapeutic settings [19]. Animal studies have demonstrated oxytocin’s critical role in maternal, sexual, feeding, and pair-bonding behaviors [20–22]. Subsequent research has supported the effects of oxytocin administration on social behaviors in humans [16, 23], as well as its involvement in a number of metabolic and homeostatic processes [24]. Exogenous intranasal and intravenous oxytocin administration was shown to reduce caloric intake in humans [25, 26] as well as body weight in dietarily induced obese rhesus monkeys through decreased food intake and increased energy expenditure and lipolysis [27]. Oxytocin has also been shown to influence the cardiovascular system, with demonstrated effects on blood pressure, heart rate, heart-rate variability, and contractility [28–31]. However, the extent of these effects and evidence in humans has been limited.

Oxytocin-pathway gene variants have been associated with features of psychotic disorders, such as social cognition and emotional processing [32, 33]. There is converging evidence that oxytocin modulates several neurotransmitter systems in the brain (e.g., dopamine, glutamate, and serotonin) that have been implicated in psychotic disorders [34]. Oxytocin administration has been shown to improve positive and negative symptoms, as well as cognitive deficits [34, 35] in patients with schizophrenia, while other studies have provided conflicting evidence [36, 37]. Despite promising initial results, some of these early findings on the effects of intranasal oxytocin have failed to replicate [38] and recent research has questioned the ability of exogenous oxytocin to influence social behavior, citing issues regarding poorly understood pharmacodynamics and potential publication bias [19, 39]. While group-level studies have provided mixed findings, it is conceivable that previously diverging results may be partly explained by different experimental approaches and individual differences in treatment response [40]. The oxytocin pathway is only one of many signaling pathways involved in psychiatric disorders, there is, however, a need to shed light on the underlying mechanisms of oxytocin signaling in humans to understand the pharmacological potential of oxytocin.

A common method for examining the relationship between oxytocin gene variants and phenotypes of interest is to use candidate gene approaches, typically investigating the oxytocin-receptor gene (*OXTR)* or *CD38*, which regulates the secretion of oxytocin [41]. Given the small influence that these genetic variations exert on analyzed traits, candidate gene studies are typically statistically underpowered to precisely detect realistic effect sizes [42]. Consequently, most studies reporting the associations between single genetic variants and behavioral phenotypes have not replicated [43]. As there are over 150 genes in the oxytocin-signaling pathway, a multivariate approach examining the cumulative polygenic signal across a gene set representing relevant biological pathways will be far more likely to detect reliable effects compared with a candidate gene approach. Therefore, to explore the role of the oxytocin system in the shared genetic liability between schizophrenia, bipolar disorder, T2D, and cardiovascular risk factors, we calculated the genetic contribution of oxytocin-pathway single-nucleotide polymorphisms (SNPs) to polygenic risk for schizophrenia and bipolar disorder, as well as T2D, to analyze and compare central and peripheral contributions and their associations with anthropometric and behavioral CVD risk factors. We applied this polygenic pathway-specific approach in a very large sample (UK Biobank) with 488,377 genotyped individuals with well-characterized anthropometric (BMI, waist-to-hip ratio, impedance measures of body composition) and behavioral CVD risk factors (diet, loneliness, and social isolation).

## Methods

### Population

The UK Biobank initiative is a large-scale biobank prospective cohort established by the UK Medical Research Council and Wellcome Trust [44]. This population-based study examines the influence of genetic and environmental factors on the occurrence of disease in participants in the age range of 40–69-years old, recruited between 2006 and 2010. The study has included approximately 500,000 participants, of which 54% are female. Participants filled out questionnaires about their lifestyle, family, diet, and medical history, in addition to providing a variety of physical measures (e.g., weight). A subset of participants also filled in a mental health questionnaire online. All participants provided signed informed consent. The UK Biobank was approved by the National Research Ethics Service North West. Specific details regarding recruitment and data collection procedures have been previously published [45]. Further details regarding genotyping and quality-control procedures for the UK Biobank are well documented [46]. The present research has been conducted using the UK Biobank Resource under Application Number 27412.

### Oxytocin-pathway SNPs

We downloaded the gene-pathway consensus database (ConsensusPathDB) from the Max Planck Institute for Molecular Genetics website (cpdb.molgen.mpg.de) [47] and extracted the approved gene names annotated to the “Oxytocin signaling pathway”. We then retrieved the genomic coordinates (‘‘chromosome_name’’, ‘start_position’’, ‘end_position’’) of the default ENSEMBL transcripts annotated to those gene names (filtering on ‘hgnc_symbol’’) using BiomaRt (host = ‘‘grch37.ensembl.org’’, path = ‘‘/biomart/martservice’’, dataset = ‘‘hsapiens_gene_ensembl’’) [48]. We complemented the transcript regions obtained with any regulatory elements annotated to the same gene names in the ORegAnno [49] database (January 2016 version) for Homo sapiens. All 1000 genomes’ phase-3 variants [50] found in the European subsample and located within the resulting genomic regions were assigned to the oxytocin-signaling pathway.

### Oxytocin-pathway polygenic score (PGS_oxt_)

We calculated three oxytocin-pathway polygenic scores (PGS_oxt_) using PRSice-2 (version 2.3.3) [51], one each for SCZ, BD, and T2D, by limiting the calculation to SNPs belonging to the oxytocin-signaling pathway in European ancestry samples, adopting a methodology similar to that of a previous report [52]. The schizophrenia PGS_oxt_ was based on a meta-analysis of the European-ancestry subset of the 2020 Psychiatric Genomics Consortium schizophrenia GWAS [53], the bipolar disorder PGS_oxt_ was based on a meta-analysis of the European-ancestry subset of the 2019 PGC bipolar disorder GWAS [54], and the T2D mellitus PGS was based on a meta-analysis of the European Caucasian subset of the DIAGRAM 2012 GWAS [55]. For each GWAS, 2000 PGS_oxt_ scores were calculated from thresholds ranging from 5 × 10^−8^ to 1, in increments of 0.001. Using a permutation approach included in PGSice-2 [51], we determined an optimal PGS threshold of *p* < 0.05 for schizophrenia (including 427 SNPs), *p* < 0.02 for bipolar disorder (including 198 SNPs), and *p* < 0.4 for T2D (including 1107 SNPs) and used PGSs computed at these thresholds for the main analysis, after a transformation to z-scores. We also mapped the SNPs included in each PGS_oxt_ (using the -print-snp flag in PRSice) using the snp2gene component of FUMA [56] and subsequently ran enrichment in DAVID [57, 58] using KEGG pathways, confirming enrichment for the oxytocin-signaling pathway. We report the list of genes (using the standard gene symbol nomenclature) and the results of the enrichment analysis (Supplementary notes1–3, Supplementary Tables 1–3) and the SNP overlap between the three PGS_oxt_ (Supplementary Fig. 1) in the supplementary materials.

### Principal components of PGS (PC–PGS)

In addition to using a series of oxytocin-pathway PGSs based on the initial threshold, for each phenotype, we also performed a principal component analysis (PCA) on the whole range of PGSs computed for each GWAS, following previously used methods [59–61] and then using the first two oxytocin-pathway PGS–principal components (PC_oxt_), as in the main analysis (PC1_oxt_ and PC2_oxt_). The first PC reweights the variants included in the PGS to achieve maximum variation over all PGS thresholds used (Supplementary Fig. 2). This unsupervised approach incorporates all computed scores across a range of tuning parameters and is agnostic regarding the outcome of interest, therefore helping control type-1 error rates [59] and capturing the greatest variation of the oxytocin-pathway PGSs computed under a range of parameter settings [59]. We included the first two components to analyze different components of polygenic association, in addition to the PGS at the empirically calculated threshold, as they would capture different signals with potentially opposite directions of effect.

### Phenotypical variables

The following metabolically relevant variables were considered (Table 1): anthropometric and cardiovascular variables (such as BMI, waist-to-hip ratio, and grip strength), body-composition variables measured through impedanciometry, and dietary-intake variables averaged from the responses to the one-day online dietary-recall questionnaire. Hand-grip strength, in particular, has shown prognostic value in a number of outcomes in adults, particularly older ones, such as physical activity, diabetes, metabolic syndrome, and cardiovascular mortality [62–64]. BMI, waist-to-hip ratio, diet, and body composition also have shown to be associated with metabolic and cardiovascular outcomes [65, 66]. In addition, several social variables were analyzed to investigate the impact on the quantity and quality of social interactions, as these have been shown to affect CVD risk [67]. Loneliness (UK Biobank variable identifier: 2020) was operationalized as a binary variable based on the question, “Do you often feel lonely?”, to which individuals answered “yes” (coded as 1) or “no” (coded as 0). The Ability to Confide (UK Biobank variable identifier: 2110) was also operationalized as a binary variable, based on the question, “How often are you able to confide in someone close to you?”. As per previous research [68], responses ranging from “almost daily” to “about once a month” were coded as “0”, and responses ranging from “once every few months” to “never” were coded as “1”. Social Contact Frequency was operationalized as a composite binary variable based on the questions, ‘Including yourself, how many people are living together in your household?’’ (UK Biobank variable identifier: 709) and “How often do you visit friends or family or have them visit you?” (UK Biobank variable identifier: 1031). Those who lived alone and who indicated that they either never visited or had no friends or family who visited were coded as “1”, and those who either did not live alone, or had friends who visited at least once a week, were coded as “0”, as done in previous research [69]. Engagement in leisure and social activities (UK Biobank variable identifier: 6160) was converted into a binary variable, determined by the response to the question, “Which of the following do you attend once a week or more often? (you can select more than one)”, with the response options “sports club or gym”, “pub or social club”, “religious group”, “adult education class”, and “other group activity”. Individuals who reported habitual participation in at least one activity were coded as “0”, all others as “1”, as reported in previous literature [69].

**Table 1:** Anthropometric and cardiovascular variables.

Variable	Observation count	UK Biobank code(s)
BMI (kg/m2)	485,323	21001
BMI estimated by impedance (kg/m2)	478,550	23104
Waist-to-hip ratio	486,124	48; 49
Grip strength (kg)	484,084	46; 47
Whole-body fat percentage	478,285	23099
Trunk fat percentage	478,235	23127
Total daily energy intake (kJ)	206,524	100002
Total daily sugar intake (g)	206,524	100008
Total daily food weight (g)	206,524	100001
Loneliness	478,140	2020
Ability to confide	469,417	2110
Social contacts	499,373	709; 1031
Participation in social activities	497,030	6160

### Statistical analysis

All statistical analyses were done using R version 4.0.0 (2020-04-24) [70]. To analyze the relationships between continuous variables, we ran pairwise correlation analyses combined with complete linkage hierarchical clustering to identify clusters of highly correlated variables. We then ran a principal component analysis within each cluster, then used the first principal component in linear regressions. We also fitted linear regression models to analyze the relationships between individual continuous variables and the PGS_oxt_’s, controlling for sex and age [2]; and tested for interaction effects by including interaction terms in the regression models with the phenotypes that clustered together with the dependent variable, as we found significant associations between those phenotypical variables. This would allow to test whether the effect of the PGS_oxt_ on a dependent phenotype changes, depending on the value of one or more other variables. For dichotomous variables, we tested their independence using Pearson’s *χ*2 test and then fitted logistic regression models, also controlling for sex and age [2] and 10 top principal components from the variance-standardized relationship matrix to account for population stratification, along with an interaction component comprising the variables that were not found to be independent. FDR correction across all tests was used to control for multiple testing. Sex was determined from genetic data. As PGSs calculated from any set of SNPs will have a nonzero effect, we compared the effect sizes of the various models against those of models using PGSs calculated from 100 random, unique gene sets of equal size to assess the specificity of the results. These distributions were often not symmetrical, possibly due to associations between the analyzed phenotype and the target phenotype for the PGS calculation. Analysis scripts to reproduce the data processing and analyses are available at https://osf.io/gpdxm/.

## Results

### Associations between cardiovascular risk-factor phenotypes

**Fig. 1:**
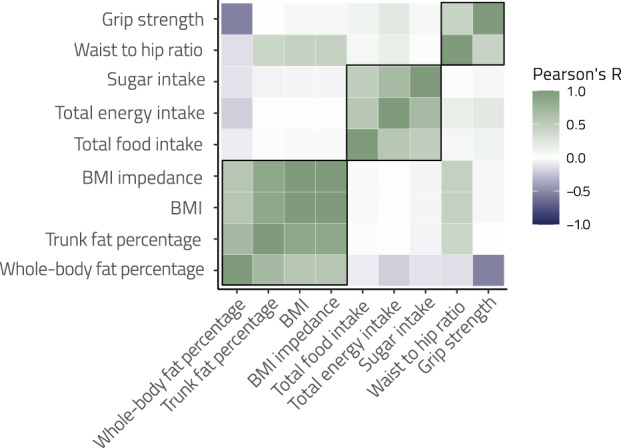
Associations between continuous phenotypes. Hierarchical analysis revealed three main clusters of associations: a BMI/body-fat cluster, a dietary cluster, and a grip strength/waist-to-hip ratio cluster. All variables within clusters were significantly associated with each other (all *p*’s < 0.05, FDR-corrected).

Pairwise correlation analyses revealed strong associations between BMI variables, trunk fat, and whole-body fat, and between grip strength and waist-to-hip ratio (Fig. 1, Supplementary Table 4). Hierarchical analysis discovered three main clusters of metabolic predictors—a BMI/body-fat cluster, a dietary cluster, and a grip strength/waist-to-hip ratio cluster. All variables within clusters had statistically significant relationships (all *p*’s < 0.05, FDR-corrected).

### Principal component analysis of continuous phenotype clusters

A principal component analysis was performed within each cluster, and the first principal component of each was then extracted. The first principal component of the BMI/body fat cluster explained 83% of the variance of that cluster, the first principal component of the dietary cluster explained 71% of the variance of that cluster, and the first principal component of the grip strength/waist-to-hip ratio cluster explained 70% of the variance within that cluster (Supplementary Fig. 3). Pairwise correlation analyses between the variables within a cluster and the first principal component of that cluster revealed strong correlation across all variables (Supplementary Fig. 3).

### Bipolar disorder oxytocin-specific polygenic scores vs CVD risk factors

**Fig. 2:**
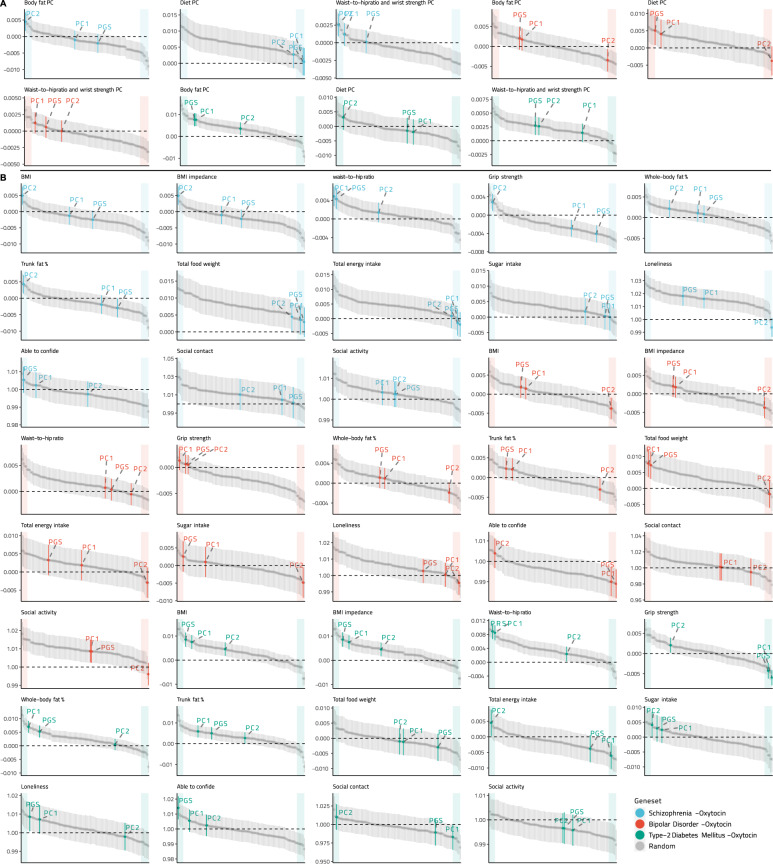
Distribution plots of the effect sizes of the PGS_oxt_ and PC–PGS_oxt_ models and models from PGSs calculated from 100 random gene sets of equal size for cluster principal components and individual variables. **A**, **B** We found that a number of the statistically significant effects are specific to the oxytocin pathway and not due to the random association of the SNP in the gene set with the chosen phenotype*s:* schizophrenia, bipolar disorder, and type-2 diabetes mellitus. PGS polygenic score, PC1 first PGS principal component, PC2 second PGS principal component.

**Fig. 3:**
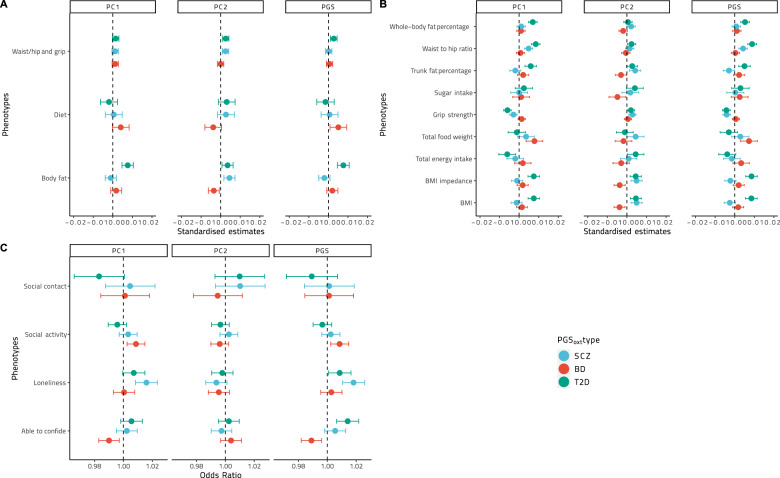
Effect sizes and confidence intervals for the linear models of the cluster principal components continuous phenotypes and logistic regression for the binary phenotypes. **A–C** Different colors correspond to the three different PGS_oxt_’s and PC–PGS_oxt_’s. BD bipolar disorder, SCZ schizophrenia, T2D type-2 diabetes mellitus, PGS_oxt_ polygenic score for the oxytocin pathway, PC1 first PGS principal component, PC2 second PGS principal component.

Models between the first principal component of each continuous-phenotype cluster and BD PGS_oxt_, BD PC1_oxt_, and BD PC2_oxt_ showed significant and pathway-specific effects (i.e., in the top or bottom 5% of the effect-size distribution for 100 random models) only between BD PC2_oxt_ and the body-fat cluster principal component (*p*_fdr_ = 0.05, Supplementary Table 5). Examining the models with the individual phenotypes, no significant association was detected between BMI and either BD PGS_oxt_ or BD PC1_oxt_ (both linear models *p*_fdr_ > 0.05), while BD PC2_oxt_ was significantly negatively associated with BMI (*p*_fdr_ = 0.026; Supplementary Tables 6–8; Figs. 2, 3) and BMI calculated from impedanciometry (*p*_fdr_ = 0.03), with these results being in the lower end of effect sizes compared with the random gene-set PGS models (Fig. 2). The models did not show any significant interaction effects between BD PC2_oxt_ and any of the other variables of the body-fat cluster (Supplementary Tables 9–10). Neither whole-body fat percentage nor trunk-fat percentage, waist-to-hip ratio, or grip strength showed significant associations with BD PGS_oxt_, BD PC1_oxt_, or BD PC2_oxt_ (all *p*_fdr_ > 0.05). Of the dietary variables, only total sugar intake was significantly negatively associated with BD PC2_oxt_ (*p*_fdr_’s < 0.001) but not BD PGS_oxt_ or BD PC1_oxt_ (both *p*_fdr_ > 0.05), with an effect size at the lower 5% of the distribution of random gene-set PGS models. The model showed significant positive interaction effects between BD PC2_oxt_ and the other two variables in the dietary cluster (Supplementary Table 11). Food weight was significantly positively associated with BD PC1_oxt_ and BD PGS_oxt_ (*p*_fdr_ < 0.001), with effect sizes in the top 5% of the random PGS model distribution, and with significant negative interaction effects between BD PGS_oxt_, PC1_oxt_, and mean sugar intake (Supplementary Tables 12, 13).

Logistic-regression models of loneliness on BD PGS_oxt_, BD PC1_oxt_, and BD PC2_oxt_ showed no significant associations (all *p*_fdr_ > 0.05; Supplementary Tables 14–16; Fig. 3). Logistic regression modeling the ability to confide showed significant negative associations with BD PGS_oxt_ and BD PC1_oxt_ (both *p*_fdr_ = 0.02 and *p*_fdr_ = 0.03, respectively) but not BD PC2_oxt_ (*p*_fdr_ > 0.5), with effect sizes at the lower 5% of the random gene-set PGS model effect size distribution. These models did not show evidence of significant interaction effects between BD PGS_oxt_ and BD PC1_oxt_ and the other social variables in their respective models (Supplementary Tables 17, 18). BD PGS_oxt_ and BD PC1_oxt_ scores showed significant positive association with participation in social activities (both *p*_fdr_’s = *0.035*), but the effect sizes were on average compared to those of the random gene-set PGS model distribution. None of the polygenic score models with the presence of social contacts showed any significant association (all *p*_fdr_ > 0.05).

### Schizophrenia oxytocin-specific polygenic scores vs CVD risk factors

Models between the first principal component of each continuous-phenotype cluster and SCZ PGS_oxt_, SCZ PC1_oxt_, and SCZ PC2_oxt_ were statistically significant and demonstrated specificity to the oxytocin pathway (i.e., in the top or bottom 5% of the effect-size distribution for 100 random models) only between the body fat cluster principal component and SCZ PC2_oxt,_ and between the waist-to-hip/grip-strength cluster principal component and SCZ PC2_oxt_ (Figs. 2, 3). Analyzing individual phenotypes, linear models demonstrated a significant positive association of BMI, BMI calculated from impedanciometry, and trunk-fat percentage with SCZ PC2_oxt_ (*p*_fdr_ < 0.005), but not with SCZ PGS_oxt_, SCZ PC1_oxt_ (both *p*_fdr_ > 0.05; Supplementary Tables 6–8; Figs. 2, 3), with all effects in the top 5% of the random gene-set PGS model distributions (Fig. 2). Of the three models, only the one including trunk-fat percentage demonstrated a significant interaction effect between SCZ PC2_oxt_ and the other variables of the body-fat cluster (Supplementary Tables 19–21). We also found no significant association between total body-fat percentage and SCZ PGS_oxt_, SCZ PC1_oxt_, and SCZ PC2_oxt_ (all *p*_*fdr*_ < 0.005). Linear-regression models of waist-to-hip ratio showed significant positive associations with SCZ PGS_oxt_ and SCZ PC1_oxt_ (both *p*_fdr_ < 0.001), but not with PGS_oxt_ or PC2_oxt_ (*p*_fdr_ > 0.05) with effect sizes in the top 5% of the random gene-set PGS model distribution (Fig. 2). Both significant models showed significant interaction effects between the respective independent variable and grip strength, the other variable in the cluster (Supplementary Tables 22–23). Significant positive associations were also found between grip strength and SCZ PGS_oxt_, SCZ PC1_oxt_, and a negative association with SCZ PC2_oxt_ (all *p*_*fdr*_ < 0.001), but only the latter had an effect size in the top or bottom 5% of the random gene-set PGS model distribution (Fig. 2), and the model showed a significant negative-interaction effect between SCZ PC2_oxt_ and waist-to-hip ratio (Supplementary Table 24). None of the models of any dietary variable showed any significant associations with SCZ PGS_oxt_, SCZ PC1_oxt_, or SCZ PC2_oxt_ (all *p*_fdr_ > 0.05).

Logistic-regression models of loneliness showed consistently significant positive associations with SCZ PGS_oxt_ and SCZ PC1_oxt_ (both *p*_fdr_’s < 0.001; Supplementary Tables 9–11; Fig. 3), but not with SCZ PC2_oxt_ (*p*_fdr_ > 0.05), though these significant effects were around the average within the random gene-set PGS model distribution. None of the other social variables showed any significant association with PGS_oxt_, SCZ PC1_oxt_, or PC2_oxt_ (all *p*_fdr_ > 0.05).

### T2D oxytocin-specific polygenic scores vs CVD risk factors

Models between the first principal component of each continuous-phenotype cluster and T2D PGS_oxt_, T2D PC1_oxt_, and T2D PC2_oxt_ did not show significant or pathway-specific effects (Supplementary Table 5). When considering individual phenotypes, linear models revealed a significant positive relationship between T2D PGS_oxt_, T2D PC1_oxt_, T2D PC2_oxt_, and BMI (all *p*_fdr_’s < 0.001; Supplementary Tables 6–8; Fig. 2), as well as BMI calculated from impedanciometry (all *p*_fdr_’s < 0.001), with these effects in the top 10% of the random gene-set PGS model distribution (Fig. 2). Models between T2D PGS_oxt_, T2D PC1_oxt_, and whole-body-fat percentage (both *p*_fdr_’s < 0.001) and trunk-fat percentage (both *p*_fdr_’s < 0.001) showed significant positive associations, but these effects are on average within the random gene-set PGS model distributions (Fig. 2). We also found significant positive relationships in models between waist-to-hip ratio and T2D PGS_oxt_, T2D PC1_oxt_, and T2D PC2_oxt_ (all *p*_fdr_’s < 0.001), with the effects of the T2D PGS_oxt_ and T2D PC1_oxt_ models in the top 5% of the random gene-set PGS model distribution, with a significant negative-interaction component between T2D PGS_oxt_ and T2D PC1_oxt_ and grip strength in each respective model (Supplementary Table 25). In addition, we found a negative association between grip strength and T2D PGS_oxt_ and T2D PC1_oxt_ (both *p*_fdr_’s < 0.001), but not T2D PC2_oxt_ (*p*_*fdr*_ > 0.05), with effect sizes in the bottom 5% of the random gene-set PGS model distributions. Both these models also showed significant negative interaction effects between waist-to-hip-ratio and T2D PGS_oxt_ and T2D PC1_oxt_, respectively (Supplementary Tables 26, 27). Of the dietary variables, only the linear model between total caloric intake, T2D PC1_oxt_ showed significant negative associations (*p*_fdr_’s = 0.01), with this effect size in the bottom 5% of the random gene-set PGS model distribution, and the model showed significant interaction effects between T2D PC1_oxt_ and the two other variables in the dietary cluster (Supplementary Table 28).

Of the social variables, only the logistic regression for the ability to confide and T2D PGS_oxt_, showed significant positive associations (*p*_fdr_ = 0.003), with an effect size at the very top of the random gene-set PGS model distribution (Fig. 2). This model did not show any significant interaction effect (Supplementary Table 29).

## Discussion

The present results derived from a sample of just under half-a-million participants suggest that the oxytocin system has pleiotropic effects on both social and metabolic phenotypes [16] by providing evidence for the involvement of the oxytocin-signaling pathway in the shared genetic liability of schizophrenia, bipolar disorder, T2D, and several phenotypes linked with CVD and T2D risk. While the effect sizes generated by these analyses were relatively small, they were comparable with those of other reports on polygenic scores [71, 72], as well as often showing specificity to the oxytocin pathway when compared with models with random gene-set PGSs (Fig. 2).

The PGS_oxt_ and PC–PGS_oxt_ were used as proxies for the oxytocin-pathway-specific liability for schizophrenia, bipolar disorder, and T2D to investigate the genetic association with CVD risk factors. While overall PGSs that combine the effect of all the SNPs associated with a specific phenotype (e.g., schizophrenia) may be better for predicting overall associations, pathway-specific PGS can be more suitable for investigating specific components of the underlying pathology [52], potentially capturing disparate associations of components of a pathway associated with different phenotypes, and have been used successfully in investigating psychotic disorders [73]. This is particularly relevant in the realm of oxytocin research, where studies have historically used candidate gene and single SNP approaches [74–76].

In the initial pairwise correlation and clustering analysis, we identified three main clusters of metabolic predictors: BMI, trunk fat, and total body fat (body-fat cluster); energy intake, sugar intake and food weight (eating-habit cluster), and grip strength and waist-to-hip ratio (strength/WH-ratio cluster). The body-fat cluster variables showed a positive association with the oxytocin-specific PGS and the first PC–PGS_oxt_ for T2D and the second PC–PGS_oxt_ for SCZ, and a negative association with the second PC–PGS_oxt_ for BD, and the body-fat cluster principal component showed a positive association with the second PC–PGS_oxt_ for SCZ. The food-intake cluster points to an involvement of the oxytocin pathway in the regulation of caloric intake, appetite, and preference for sweet food, consistent with previous reports [18]. While the relationship between grip strength and waist-to-hip ratio (grip strength/WH-ratio cluster) is less expected than the other clusters, both these highly related markers have prognostic value for cardiovascular events and mortality [62, 77].

Four variables were included to investigate the role of oxytocin-pathway variants on social behavior: loneliness, the ability to confide, social contacts, and social activity. Loneliness and the ability to confide represent the subjective dissatisfaction with one’s relationships, with the ability to confide significantly associated with the oxytocin-pathway PGSs. While the level of social contacts was strongly linked with loneliness, it was not associated with most of the oxytocin-specific PRSs, and while these variables were found not to be independent, the logistic regression models found no significant interactions between them. These associations with the oxytocin pathway are in line with previous findings on emotional withdrawal in individuals with schizophrenia [32] and on the symptomatology of individuals with bipolar disorder [78, 79]. In other words, although many participants in the present sample reported that they had very little social contact, this was not necessarily *distressing* for all these individuals, and oxytocin-pathway variants were found to be associated with reported dissatisfaction with the quantity or quality of social contact.

It has recently been proposed that the primary purpose of the oxytocin-signaling system is the support of allostasis, which is the process of maintaining stability in changing environments [24]. This is a departure from existing theories of oxytocin’s purpose that focus on its social effects [80], however, it better reflects the emerging literature demonstrating its nonsocial effects and its evolutionary history [24]. Organism survival depends on efficient energy regulation, which is facilitated by adjusting behaviors based on current and predicted environmental conditions. For humans, an important element of predicting future environmental conditions is understanding the thoughts and intentions of other people. For example, the distinction between facial expression cues that can reveal whether someone else is going to help or harm can be very subtle. Oxytocin-like peptides first emerged around 600 million years ago [81], facilitating muscle contraction, locomotion, and food-related learning [82] in the service of energy regulation and reproduction. The Allostatic Theory of Oxytocin suggests that oxytocin signaling adapted over time to include the coordination of social behavior to help support energy-regulation processes, which is consistent with the present results regarding energy intake and body composition.

There has been a growing interest in oxytocin’s role in behavior [17]. However, the relationship between oxytocin’s behavioral and physical effects, and the implications for its behavioral effects are seldom discussed [17]. The present study adds to increasing genetic evidence that the oxytocin system is associated with a broad range of mental and somatic effects [83], and that the function of the oxytocin system has a pleiotropic influence on both these groups of phenotypes. It is conceivable that part of this observed genetic pleiotropy could be explained by population stratification and nonrandom sample selection in the original GWAS, for instance, due to varying exclusion criteria related to somatic conditions for patients and healthy controls. Another potential limitation is the lack of a sex-disaggregated PGS that would better account for sex-specific confounders [84], which is relevant for oxytocin research [17]. In addition, this methodology cannot explain the biological mechanisms that underlie the relationships found, as PGSs assume an additive effect of individual risk alleles and do not model higher-order relationships between risk variants. One last consideration must be noted with regard to the variables pertaining to social relations—the questions asked and coding of the answers are by necessity reductive of their respective phenomena and must be noted as a limitation of this study. These variables and questionnaires, however, have been used in multiple studies [85] and have been previously validated as reliable indicators of the quality and quantity of social relationships [86].

Altogether, the shared effect of oxytocin-system dysfunction helps provide a better understanding of why social and metabolic dysfunction often co-occurs in severe mental illnesses. Future investigations examining the effects of long-term effects of intranasal oxytocin administration on behavior and cognition in severe mental illnesses, such as schizophrenia, should also explore oxytocin’s effects on metabolic measures in tandem.

## Supplementary information

Supplementary materials

## Data Availability

The UK biobank is open for eligible researchers upon application (http://www.ukbiobank.ac.uk/register-apply/).

## References

[1] Chang C-K, Hayes RD, Perera G, Broadbent MTM, Fernandes AC, Lee WE (2011). Life Expectancy at Birth for People with Serious Mental Illness and Other Major Disorders from a Secondary Mental Health Care Case Register in London. PLOS ONE.

[2] Weye N, Momen NC, Christensen MK, Iburg KM, Dalsgaard S, Laursen TM (2020). Association of Specific Mental Disorders With Premature Mortality in the Danish Population Using Alternative Measurement Methods. JAMA Netw Open.

[3] Gladigau EL, Fazio TN, Hannam JP, Dawson LM, Jones SG (2014). Increased cardiovascular risk in patients with severe mental illness. Intern Med J.

[4] Vancampfort D, Stubbs B, Mitchell AJ, De Hert M, Wampers M, Ward PB (2015). Risk of metabolic syndrome and its components in people with schizophrenia and related psychotic disorders, bipolar disorder and major depressive disorder: a systematic review and meta-analysis. World Psychiatry: Off J World Psychiatr Assoc (WPA).

[5] Alberti KGMM, Zimmet P, Shaw J (2005). The metabolic syndrome—a new worldwide definition. Lancet.

[6] Birkenaes AB, Opjordsmoen S, Brunborg C, Engh JA, Jonsdottir H, Ringen PA (2007). The level of cardiovascular risk factors in bipolar disorder equals that of schizophrenia: a comparative study. J Clin psychiatry.

[7] Mitchell AJ, Vancampfort D, Sweers K, Van Winkel R, Yu W, De HM, et al. Prevalence of metabolic syndrome and metabolic abnormalities in schizophrenia and related disorders-a systematic review and meta-analysis. Schizophrenia Bull. 2013;39:306–18. 10.1093/schbul/sbr148 PMC3576174 22207632

[8] Daumit GL, Goldberg RW, Anthony C, Dickerson F, Brown CH, Kreyenbuhl J (2005). Physical Activity Patterns in Adults With Severe Mental Illness. J Nerv Ment Dis.

[9] Fontaine KR, Heo M, Harrigan EP, Shear CL, Lakshminarayanan M, Casey DE (2001). Estimating the consequences of anti-psychotic induced weight gain on health and mortality rate. Psychiatry Res.

[10] Venkatasubramanian G, Chittiprol S, Neelakantachar N, Naveen MN, Thirthall J, Gangadhar BN (2007). Insulin and insulin-like growth factor-1 abnormalities in antipsychotic-naive schizophrenia. Am J Psychiatry.

[11] Raphael T, Parsons JP (1921). Blood sugar studies in dementia praecox and manic depressive insanity. Arch Neurol Psychiatry.

[12] Fernandez-Egea E, Bernardo M, Parellada E, Justicia A, Garcia-Rizo C, Esmatjes E (2008). Glucose abnormalities in the siblings of people with schizophrenia. Schizophrenia Res.

[13] Andreassen OA, McEvoy LK, Thompson WK, Wang Y, Reppe S, Schork AJ (2014). Identifying common genetic variants in blood pressure due to polygenic pleiotropy with associated phenotypes. Hypertension (Dallas, Tex: 1979).

[14] Morgan VA, Waterreus A, Carr V, Castle D, Cohen M, Harvey C (2017). Responding to challenges for people with psychotic illness: Updated evidence from the Survey of High Impact Psychosis. Aust N Z J Psychiatry.

[15] Holt-Lunstad J, Smith TB (2016). Loneliness and social isolation as risk factors for CVD: implications for evidence-based patient care and scientific inquiry. Heart Br Card Soc.

[16] Quintana DS, Dieset I, Elvsåshagen T, Westlye LT, Andreassen OA (2017). Oxytocin system dysfunction as a common mechanism underlying metabolic syndrome and psychiatric symptoms in schizophrenia and bipolar disorders. Front Neuroendocrinol.

[17] Winterton A, Westlye LT, Steen NE, Andreassen OA, Quintana DS. Improving the precision of intranasal oxytocin research. Nat Hum Behav 1–10 (2020). 10.1038/s41562-020-00996-4. 10.1038/s41562-020-00996-4 33257880

[18] Leng G, Sabatier N (2017). Oxytocin—the sweet hormone?. Trends Endocrinol Metab.

[19] Alvares GA, Quintana DS, Whitehouse AJO (2016). Beyond the hype and hope: critical considerations for intranasal oxytocin research in autism spectrum disorder. Autism Res.

[20] Carter CS, Williams JR, Witt DM, Insel TR (1992). Oxytocin and social bonding A. Ann N. Y Acad Sci.

[21] Insel TR, Winslow JT, Wang Z, Young LJ. Oxytocin, vasopressin, and the neuroendocrine basis of pair bond formation. In Zingg HH, Bourque CW, Bichet DG, editors. Vasopressin and oxytocin. Boston, MA: Springer; 1998. p. 215–24. 10.1007/978-1-4615-871-3_28. 10.1007/978-1-4615-4871-3_28 10026808

[22] Carter CS (1992). Oxytocin and sexual behavior. Neurosci Biobehav Rev.

[23] Guastella AJ, MacLeod C (2012). A critical review of the influence of oxytocin nasal spray on social cognition in humans: evidence and future directions. Hormones Behav.

[24] Quintana DS, Guastella AJ (2020). An allostatic theory of oxytocin. Trends Cogn Sci.

[25] Ott V, Finlayson G, Lehnert H, Heitmann B, Heinrichs M, Born J (2013). Oxytocin Reduces Reward-Driven Food Intake in Humans. Diabetes.

[26] Lawson EA, Marengi DA, DeSanti RL, Holmes TM, Schoenfeld DA, Tolley CJ (2015). Oxytocin reduces caloric intake in men. Obes (Silver Spring).

[27] Blevins JE, Graham JL, Morton GJ, Bales KL, Schwartz MW, Baskin DG (2015). Chronic oxytocin administration inhibits food intake, increases energy expenditure, and produces weight loss in fructose-fed obese rhesus monkeys. Am J Physiol Regulatory, Integr Comp Physiol.

[28] Gutkowska J, Jankowski M, Mukaddam-Daher S, McCann SM (2000). Oxytocin is a cardiovascular hormone. Braz J Med Biol Res Rev Bras Pesqui Medicas E Biol.

[29] Gimpl G, Farenholtz F, Fahrenholz F, Gene C (2001). The oxytocin receptor system: structure, function and regulation. Physiol Rev.

[30] Petersson M Cardiovascular effects of oxytocin. In Poulain D, Oliet S, Theodosis D, editors. Progress in Brain Research. Elsevier; 2002. p. 281–8. 10.1016/s0079-6123(02)39024-1 12436943

[31] Kemp AH, Quintana DS, Kuhnert R-L, Griffiths K, Hickie IB, Guastella AJ (2012). Oxytocin Increases Heart Rate Variability in Humans at Rest: Implications for Social Approach-Related Motivation and Capacity for Social Engagement. PLoS ONE.

[32] Haram M, Tesli M, Bettella F, Djurovic S, Andreassen OA, Melle I. Association between genetic variation in the oxytocin receptor gene and emotional withdrawal, but not between oxytocin pathway genes and diagnosis in psychotic disorders. Front Hum Neurosci. 2015;9:9. 10.3389/fnhum.2015.00009 PMC4303871 25667571

[33] Montag C, Brockmann E-M, Bayerl M, Rujescu D, Müller DJ, Gallinat J (2013). Oxytocin and oxytocin receptor gene polymorphisms and risk for schizophrenia: A case–control study. World J Biol Psychiatry.

[34] Shilling PD, Feifel D (2016). Potential of oxytocin in the treatment of schizophrenia. CNS Drugs.

[35] Lee MR, Wehring HJ, McMahon RP, Linthicum J, Cascella N, Liu F (2013). Effects of adjunctive intranasal oxytocin on olfactory identification and clinical symptoms in schizophrenia: results from a randomized double blind placebo controlled pilot study. Schizophrenia Res.

[36] Cacciotti-Saija C, Langdon R, Ward PB, Hickie IB, Scott EM, Naismith SL (2015). A double-blind randomized controlled trial of oxytocin nasal spray and social cognition training for young people with early psychosis. Schizophr Bull.

[37] Jarskog LF, Pedersen CA, Johnson JL, Hamer RM, Rau SW, Elliott T (2017). A 12-week randomized controlled trial of twice-daily intranasal oxytocin for social cognitive deficits in people schizophrenia. Schizophr Res.

[38] Declerck CH, Boone C, Pauwels L, Vogt B, Fehr E (2020). A registered replication study on oxytocin and trust. Nat Hum Behav.

[39] Walum H, Waldman ID, Young LJ (2016). Statistical and methodological considerations for the interpretation of intranasal oxytocin studies. Biol Psychiatry.

[40] Quintana DS, Guastella AJ, Westlye LT, Andreassen OA (2016). The promise and pitfalls of intranasally administering psychopharmacological agents for the treatment of psychiatric disorders. Mol Psychiatry.

[41] Feldman R, Monakhov M, Pratt M, Ebstein RP (2015). Oxytocin pathway genes: evolutionary ancient system impacting on human affiliation, sociality, and psychopathology. Biol Psychiatry.

[42] Harden KP (2021). “Reports of my death were greatly exaggerated”: behavior genetics in the postgenomic era. Annu Rev Psychol.

[43] Harden KP, Koellinger PD (2020). Using genetics for social science. Nat Hum Behav.

[44] Sudlow C, Gallacher J, Allen N, Beral V, Burton P, Danesh J (2015). UK Biobank: An Open Access Resource for Identifying the Causes of a Wide Range of Complex Diseases of Middle and Old Age. PLOS Med.

[45] UK Biobank. UK Biobank: protocol for a large-scale prospective epidemiological resource. 2007. https://www.ukbiobank.ac.uk/media/gnkeyh2q/study-rationale.pdf

[46] Bycroft C, Freeman C, Petkova D, Band G, Elliott LT, Sharp K (2018). The UK Biobank resource with deep phenotyping and genomic data. Nature.

[47] Kamburov A, Stelzl U, Lehrach H, Herwig R (2013). The ConsensusPathDB interaction database: 2013 update. Nucleic Acids Res.

[48] Durinck S, Spellman PT, Birney E, Huber W (2009). Mapping identifiers for the integration of genomic datasets with the R/Bioconductor package biomaRt. Nat Protoc.

[49] Lesurf R, Cotto KC, Wang G, Griffith M, Kasaian K, Jones SJM (2016). ORegAnno 3.0: a community-driven resource for curated regulatory annotation. Nucleic Acids Res.

[50] Auton A, Abecasis GR, Altshuler DM, Durbin RM, Abecasis GR, Bentley DR (2015). A global reference for human genetic variation. Nature.

[51] Euesden J, Lewis CM, O’Reilly PF (2015). PRSice: polygenic risk score software. Bioinformatics.

[52] Darst BF, Koscik RL, Racine AM, Oh JM, Krause RA, Carlsson CM (2017). Pathway-specific polygenic risk scores as predictors of β-amyloid deposition and cognitive function in a sample at increased risk for Alzheimer’s disease. J Alzheimers Dis.

[53] Schizophrenia Working Group of the Psychiatric Genomics ConsortiumRipke, S, Walters, JT & O’Donovan, MC Mapping genomic loci prioritises genes and implicates synaptic biology in schizophrenia. *medRxiv*. 2020. 10.1101/2020.09.12.20192922.

[54] Stahl EA, Breen G, Forstner AJ, McQuillin A, Ripke S, Trubetskoy V (2019). Genome-wide association study identifies 30 loci associated with bipolar disorder. Nat Genet.

[55] Morris AP, Voight BF, Teslovich TM, Ferreira T, Segrè AV, Steinthorsdottir V (2012). Large-scale association analysis provides insights into the genetic architecture and pathophysiology of type 2 diabetes. Nat Genet.

[56] Watanabe K, Taskesen E, van Bochoven A, Posthuma D (2017). Functional mapping and annotation of genetic associations with FUMA. Nat Commun.

[57] Huang DW, Sherman BT, Lempicki RA (2009). Bioinformatics enrichment tools: paths toward the comprehensive functional analysis of large gene lists. Nucleic Acids Res.

[58] Huang DW, Sherman BT, Lempicki RA (2009). Systematic and integrative analysis of large gene lists using DAVID bioinformatics resources. Nat Protoc.

[59] Coombes BJ, Ploner A, Bergen SE, Biernacka JM (2020). A principal component approach to improve association testing with polygenic risk scores. Genet Epidemiol.

[60] Alnæs D, Kaufmann T, van der Meer D, Córdova-Palomera A, Rokicki J, Moberget T (2019). Brain Heterogeneity in Schizophrenia and Its Association With Polygenic Risk. JAMA Psychiatry.

[61] de Lange A-MG, Kaufmann T, van der Meer D, Maglanoc LA, Alnæs D, Moberget T (2019). Population-based neuroimaging reveals traces of childbirth in the maternal brain. Proc Natl Acad Sci USA.

[62] Leong DP, Teo KK, Rangarajan S, Lopez-Jaramillo P, Avezum A, Orlandini A (2015). Prognostic value of grip strength: findings from the Prospective Urban Rural Epidemiology (PURE) study. Lancet.

[63] Lawman HG, Troiano RP, Perna FM, Wang C-Y, Fryar CD, Ogden CL (2016). Associations of Relative Handgrip Strength and Cardiovascular Disease Biomarkers in U.S. Adults, 2011-2012. Am J Preventive Med.

[64] Kim Y, Wijndaele K, Lee D, Sharp SJ, Wareham N, Brage S (2017). Independent and joint associations of grip strength and adiposity with all-cause and cardiovascular disease mortality in 403,199 adults: the UK Biobank study. Am J Clin Nutr.

[65] Dalton M, Cameron AJ, Zimmet PZ, Shaw JE, Jolley D, Dunstan DW (2003). Waist circumference, waist-hip ratio and body mass index and their correlation with cardiovascular disease risk factors in Australian adults. J Intern Med.

[66] Franssen FME, Rutten EPA, Groenen MTJ, Vanfleteren LE, Wouters EFM, Spruit MA (2014). New Reference Values for Body Composition by Bioelectrical Impedance Analysis in the General Population: Results From the UK Biobank. J Am Med Dir Assoc.

[67] Valtorta NK, Kanaan M, Gilbody S, Ronzi S, Hanratty B (2016). Loneliness and social isolation as risk factors for coronary heart disease and stroke: systematic review and meta-analysis of longitudinal observational studies. Heart.

[68] Elovainio M, Hakulinen C, Pulkki-Råback L, Virtanen M, Josefsson K, Jokela M (2017). Contribution of risk factors to excess mortality in isolated and lonely individuals: an analysis of data from the UK Biobank cohort study. Lancet Public Health.

[69] Day FR, Ong KK, Perry JRB (2018). Elucidating the genetic basis of social interaction and isolation. Nat Commun.

[70] R Core Team. R: A Language and Environment for Statistical Computing. R Foundation for Statistical Computing; 2020.

[71] Begum F, Ghosh D, Tseng GC, Feingold E (2012). Comprehensive literature review and statistical considerations for GWAS meta-analysis. Nucleic Acids Res.

[72] Dudbridge F (2013). Power and predictive accuracy of polygenic risk scores. PLoS Genet.

[73] Holland JF, Cosgrove D, Whitton L, Harold D, Corvin A, Gill M (2020). Effects of complement gene-set polygenic risk score on brain volume and cortical measures in patients with psychotic disorders and healthy controls. Am J Med Genet Part B: Neuropsychiatr Genet.

[74] Poore HE, Waldman ID (2020). The association of oxytocin receptor gene (OXTR) polymorphisms antisocial behavior: a meta-analysis. Behav Genet.

[75] Baribeau DA, Dupuis A, Paton TA, Scherer SW, Schachar RJ, Arnold PD (2017). Oxytocin Receptor Polymorphisms are Differentially Associated with Social Abilities across Neurodevelopmental Disorders. Sci Re.

[76] Uzefovsky F, Bethlehem RAI, Shamay-Tsoory S, Ruigrok A, Holt R, Spencer M (2019). The oxytocin receptor gene predicts brain activity during an emotion recognition task in autism. Mol Autism.

[77] de Koning L, Merchant AT, Pogue J, Anand SS (2007). Waist circumference and waist-to-hip ratio as predictors of cardiovascular events: meta-regression analysis of prospective studies. Eur Heart J.

[78] Turan T, Uysal C, Asdemir A, Kılıç E (2013). May oxytocin be a trait marker for bipolar disorder?. Psychoneuroendocrinol.

[79] Wei S-Y, Tseng H-H, Chang HH, Lu T-H, Chang WH, Chiu NT (2020). Dysregulation of oxytocin and dopamine in the corticostriatal circuitry in bipolar II disorder. Transl Psychiatry.

[80] Shamay-Tsoory SG, Abu-Akel A (2016). The social salience hypothesis of oxytocin. Biol Psychiatry.

[81] Gwee P-C, Tay B-H, Brenner S, Venkatesh B (2009). Characterization of the neurohypophysial hormone gene loci in elephant shark and the Japanese lamprey: origin of the vertebrate neurohypophysial hormone genes. BMC Evol Biol.

[82] Beets I, Janssen T, Meelkop E, Temmerman L, Suetens N, Rademakers S (2012). Vasopressin/oxytocin-related signaling regulates gustatory associative learning in C. elegans. Science.

[83] Quintana DS, Rokicki J, van der Meer D, Alnæs D, Kaufmann T, Córdova-Palomera A (2019). Oxytocin pathway gene networks in the human brain. Nat Commun.

[84] Flynn E, Tanigawa Y, Rodriguez F, Altman RB, Sinnott-Armstrong N, Rivas MA (2020). Sex-specific genetic effects across biomarkers. Eur J Hum Genet.

[85] Hakulinen C, Pulkki-Råback L, Virtanen M, Jokela M, Kivimäki M, Elovainio M (2018). Social isolation and loneliness as risk factors for myocardial infarction, stroke and mortality: UK Biobank cohort study of 479,054 men and women. Heart.

[86] Cacioppo JT, Hughes ME, Waite LJ, Hawkley LC, Thisted RA (2006). Loneliness as a specific risk factor for depressive symptoms: cross-sectional and longitudinal analyses. Psychol Aging.

