# Supplementary material for: Oxytocin-pathway polygenic scores for severe mental disorders and metabolic phenotypes in the UK Biobank
Source: Transl Psychiatry. 2021 Nov 25;11:599. doi: 10.1038/s41398-021-01725-9 (PMC8616952; doi:10.1038/s41398-021-01725-9)
Supplement: Supplementary file 1 — Supplementary materials [file 41398_2021_1725_MOESM1_ESM.pdf]

Oxytocin pathway polygenic scores for severe mental disorder and metabolic phenotypes in the UK Biobank

Supplementary materials

Supplementary figure 1 – SNP overlap between the three PGSoxt

Supplementary figure 2 – Scree plot of the 3 PC-PRS

Supplementary figure 3 – Scree plot and correlation matrices for the continuous variable clusters

Supplementary note 1 – Gene list for the SCZ PGS_{oxt}

Supplementary note 2 – Gene list for the BD PGS_{oxt}

Supplementary note 3 – Gene list for the T2D PGS_{oxt}

Supplementary table 1 – Gene enrichment for the SCZ PGS_{oxt}

Supplementary table 2 – Gene enrichment for the BD PGS_{oxt}

Supplementary table 3 – Gene enrichment for the T2D PGS_{oxt}

Supplementary table 4 – Correlation matrix of continuous variables

Supplementary table 5 – Linear PRS model results for the continuous variable cluster PCs

Supplementary table 6 – Linear PRS models results for continuous phenotypes

Supplementary table 7 – Linear PC1 models results for continuous phenotypes

Supplementary table 8 – Linear PC2 models results for continuous phenotypes

Supplementary table 9 – Interactions BMI ~ BD PGS_{oxt} PC2

Supplementary table 10 – Interactions BMI (impedance) ~ BD PGS_{oxt} PC2

Supplementary table 11 – InteractionsSugar intake ~ BDPGSoxtPC2

Supplementary table 12 – Interactions Food weight ~ BD PGSoxt

Supplementary table 13 – Interactions Food weight ~ BD PGS_{oxt} PC

Supplementary table 14 – Logistic regression PRS models results for dichotomous phenotypes

Supplementary table 15 – Logistic regression PC1 models results for dichotomous phenotypes

Supplementary table 16 – Logistic regression PC2 models results for dichotomous phenotypes

Supplementary table 17 – Interactions Ability to confide ~ BD PGS_{oxt}

Supplementary table 18 – Interactions Ability to confide ~ BD PGS_{oxt} PC

Supplementary table 19 – Interactions BMI ~ SCZ PGS_{oxt} PC2

Supplementary table 20 – Interactions BMI (impedance) ~ SCZ PGS_{oxt} PC2

Supplementary table 21 – Interactions Trunk fat percentage ~ SCZ PGS_{oxt} PC2

Supplementary table 22 – Interactions Waist-to-hip ratio ~ BD PGS_{oxt}

Supplementary table 23 – Interactions Waist-to-hip ratio ~ BD PGS_{oxt} PC

Supplementary table 24 – Interactions Grip strength ~ BD PGS_{oxt} PC2

Supplementary table 25 – Interactions Waist-to-hip ratio ~ T2D PGS_{oxt} PC

Supplementary table 26 – Interactions Grip strength ~ T2D PGS_{oxt}

Supplementary table 27 – Interactions Grip strength ~ T2D PGS_{oxt} PC2

Supplementary table 28 – Interactions Energy intake ~T2D PGS_{oxt} PC

Supplementary table 29 – Interactions Ability to confide ~T2D PGS_{oxt}

Supplementary Figure 1 – SNP overlap between the three PGS_{oxt}

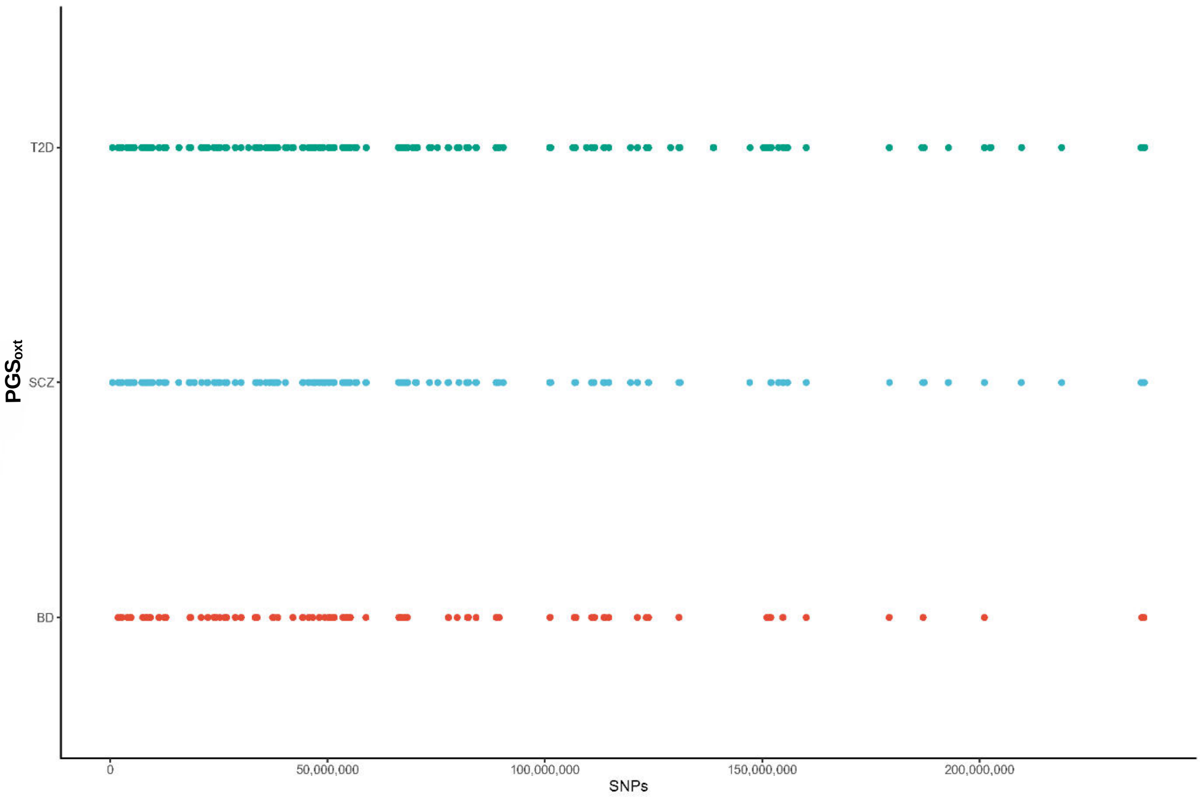

Supplementary Figure 2 – Scree plots of the 3 PC-PGS procedures

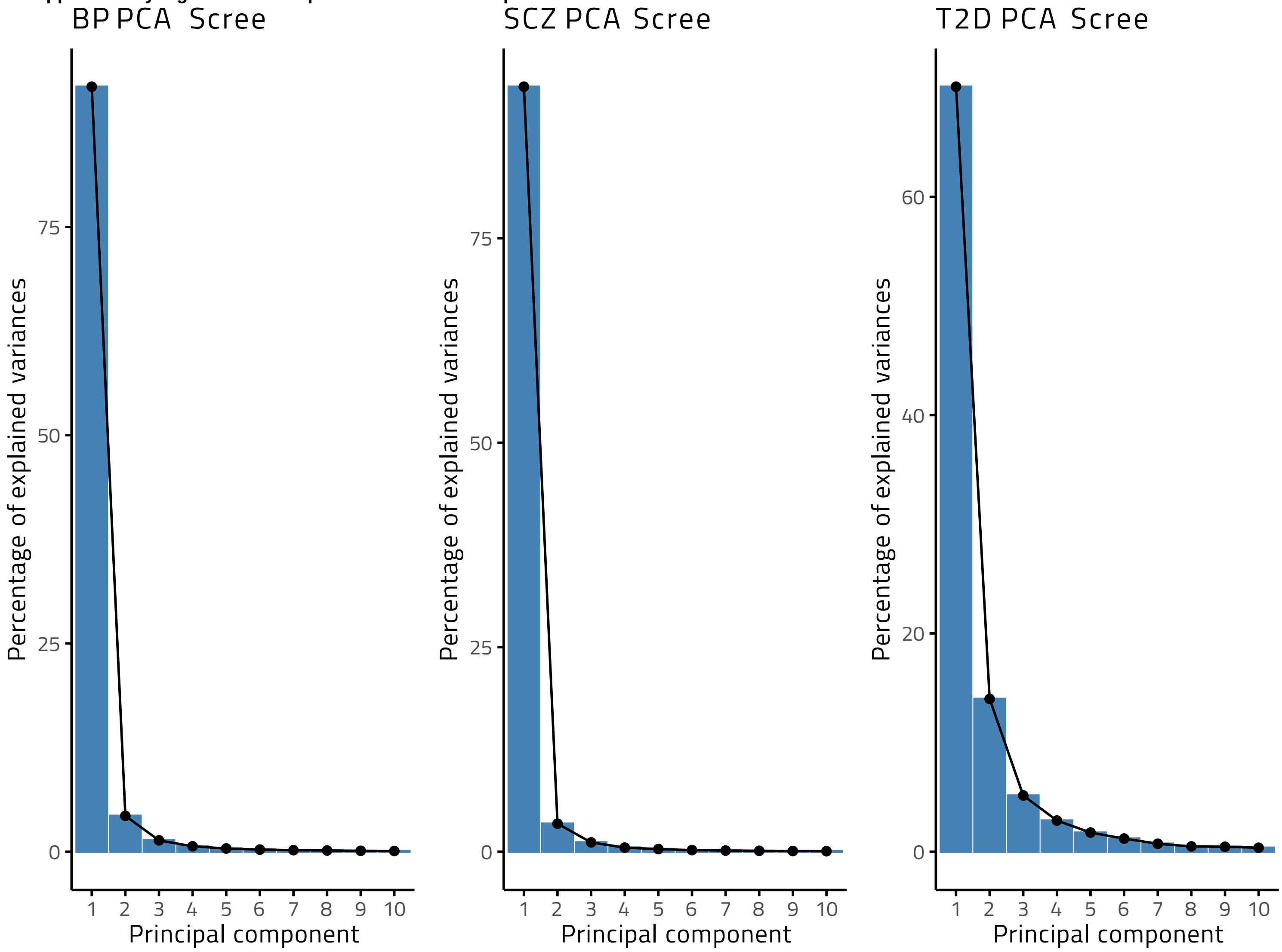

Supplementary Figure 3 – Scree plots and correlation matrices of the continuous variable clusters

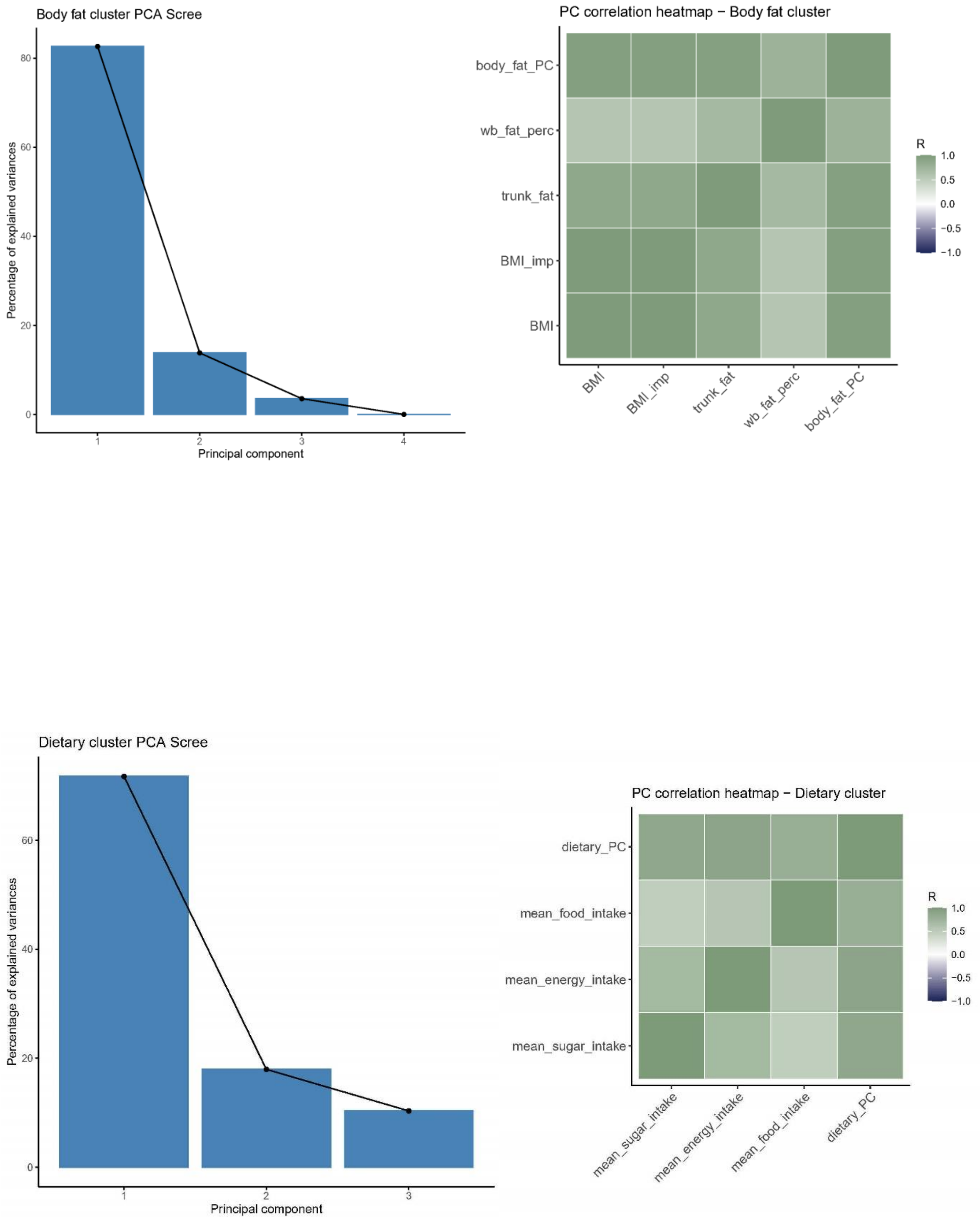

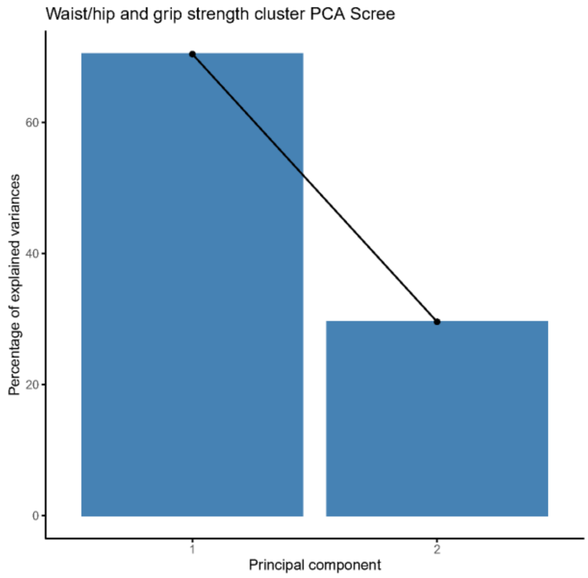
Waist/hip and grip strength cluster PCA Scree
Percentage of explained variances
0
20
40
60
1
2
Principal component

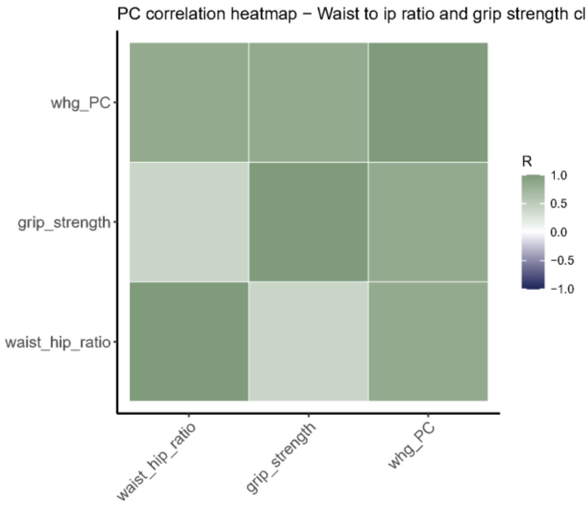
PC correlation heatmap − Waist to ip ratio and grip strength cl
whg_PC
grip_strength
waist_hip_ratio
waist_hip_ratio
grip_strength
whg_PC
R
1.0
0.5
0.0
−0.5
−1.0

Supplementary Note 1 – SCZ PGS GENE list

ADCY2, BAK1, ITPR3, UQCC2, IP6K3, LEMD2, PCLO, CACNA1C, ITFG2, SMAD3, AAGAB, IQCH, C15orf61, MAP2K5, SKOR1, CLN6, USP31, PRKCB, KIAA0895L, EXOC3L1, E2F4, FHOD1, PLEKHG4, KCTD19, LRRC36, TPPP3, ZDHHC1, HSD11B2, ATP6V0D1, AGRP, FAM65A, CTCF, RLTPR, ACD, PARD6A, C16orf86, GFOD2, RANBP10, TSNAXIP1, CENPT, NUTF2, EDC4, NRN1L, PSKH1, CTRL, CTC-479C5.12, PSMB10, LCAT, SLC12A4, DPEP3, DPEP2, DUS2, DDX28, NFATC3, ESRP2, PLA2G15, SLC7A6, SLC7A6OS, PRMT7, SMPD3, ARHGAP40, ACTR5, PPP1R16B, FAM83D, DHX35.

Supplementary Note 2 – BD PGS GENE list

ADCY2, BAK1, ITPR3, UQCC2, IP6K3, LEMD2, PCLO, CACNA1C, ITFG2, SMAD3, AAGAB, IQCH, C15orf61, MAP2K5, SKOR1, CLN6, USP31, PRKCB, KIAA0895L, EXOC3L1, E2F4, FHOD1, PLEKHG4, KCTD19, LRRC36, TPPP3, ZDHHC1, HSD11B2, ATP6V0D1, AGRP, FAM65A, CTCF, RLTPR, ACD, PARD6A, C16orf86, GFOD2, RANBP10, TSNAXIP1, CENPT, NUTF2, EDC4, NRN1L, PSKH1, CTRL, CTC-479C5.12, PSMB10, LCAT, SLC12A4, DPEP3, DPEP2, DUS2, DDX28, NFATC3, ESRP2, PLA2G15, SLC7A6, SLC7A6OS, PRMT7, SMPD3, ARHGAP40, ACTR5, PPP1R16B, FAM83D, DHX35.

Supplementary Note 3– T2D PGS GENE list

TAPBP, ZBTB22, DAXX, BAK1, ITPR3, UQCC2, IP6K3, LEMD2, NACAD, ADCY1, PCLO, FAM49B, CACNB2, MALRD1, WNT5B, ADIPOR2, CACNA2D4, CACNA1C, ITFG2, CBFB, TRADD, KIAA0895L, EXOC3L1, ELMO3, LRRC29, FHOD1, SLC9A5, PLEKHG4, KCTD19, LRRC36, ZDHHC1, HSD11B2, ATP6V0D1, AGRP, FAM65A, CTCF, RLTPR, ACD, PARD6A, ENKD1, C16orf86, GFOD2, RANBP10, TSNAXIP1, CENPT, THAP11, NUTF2, EDC4, NRN1L, PSKH1, CTRL, CTC-479C5.12, PSMB10, LCAT, SLC12A4, DPEP3, DPEP2, DUS2, DDX28, NFATC3, ESRP2, PLA2G15, SLC7A6, SLC7A6OS, PRMT7, SMPD3, ZFP90, CDH1, TANGO6, ARHGAP40, ACTR5, PPP1R16B, FAM83D.

SUPPLEMENTARY TABLE 1. SCZ PGS GENE ENRICHMENT

KEGG	Genes	Fold Enrichment	FDR
HSA04921:OXYTOCIN SIGNALING PATHWAY	CACNB2, NFATC3, ITPR3, CACNA2D4, CACNA1C, ADCY1	10.58307692	0.016533875
HSA05414:DILATED CARDIOMYOPATHY	CACNB2, CACNA2D4, CACNA1C, ADCY1	12.5989011	0.110122319
HSA04911:INSULIN SECRETION	PCLO, ITPR3, CACNA1C, ADCY1	12.45067873	0.110122319
HSA04010:MAPK SIGNALING PATHWAY	CACNB2, DAXX, NFATC3, CACNA2D4, CACNA1C	5.228792946	0.252582164
HSA04261:ADRENERGIC SIGNALING IN CARDIOMYOCYTES	CACNB2, CACNA2D4, CACNA1C, ADCY1	7.668896321	0.252582164
HSA04022:CGMP-PKG SIGNALING PATHWAY	NFATC3, ITPR3, CACNA1C, ADCY1	6.698149951	0.275895042
HSA04720:LONG-TERM POTENTIATION	ITPR3, CACNA1C, ADCY1	12.02622378	0.275895042
HSA05412:ARRHYTHMOGENIC RIGHT VENTRICULAR CARDIOMYOPATHY (ARVC)	CACNB2, CACNA2D4, CACNA1C	11.8467279	0.275895042
HSA04920:ADIPOCYTOKINE SIGNALING PATHWAY	TRADD, AGRP, ADIPOR2	11.33901099	0.275895042
HSA04260:CARDIAC MUSCLE CONTRACTION	CACNB2, CACNA2D4, CACNA1C	10.58307692	0.275895042
HSA05410:HYPERTROPHIC CARDIOMYOPATHY (HCM)	CACNB2, CACNA2D4, CACNA1C	10.1760355	0.275895042
HSA04925:ALDOSTERONE SYNTHESIS AND SECRETION	ITPR3, CACNA1C, ADCY1	9.799145299	0.275895042
HSA04912:GNRH SIGNALING PATHWAY	ITPR3, CACNA1C, ADCY1	8.722316145	0.294950189
HSA04972:PANCREATIC SECRETION	CTRL, ITPR3, ADCY1	8.534739454	0.294950189
HSA04713:CIRCADIAN ENTRAINMENT	ITPR3, CACNA1C, ADCY1	8.355060729	0.294950189
HSA04723:RETROGRADE ENDOCANNABINOID SIGNALING	ITPR3, CACNA1C, ADCY1	7.858720487	0.308655949
HSA04725:CHOLINERGIC SYNAPSE	ITPR3, CACNA1C, ADCY1	7.150727651	0.337253376
HSA04724:GLUTAMATERGIC SYNAPSE	ITPR3, CACNA1C, ADCY1	6.962550607	0.337253376
HSA04270:VASCULAR SMOOTH MUSCLE CONTRACTION	ITPR3, CACNA1C, ADCY1	6.784023669	0.337253376

SUPPLEMENTARY TABLE 2. BD PGS GENE ENRICHMENT

KEGG	Genes	Fold Enrichment	FDR
HSA04921:OXYTOCIN SIGNALING PATHWAY	PRKCB, NFATC3, ADCY2, ITPR3, CACNA1C, MAP2K5	13.10285714	0.00416153
HSA04911:INSULIN SECRETION	PCLO, PRKCB, ADCY2, ITPR3, CACNA1C	19.26890756	0.00416153
HSA04925:ALDOSTERONE SYNTHESIS AND SECRETION	PRKCB, ADCY2, ITPR3, CACNA1C	16.17636684	0.032241953
HSA04540:GAP JUNCTION	PRKCB, ADCY2, ITPR3, MAP2K5	14.88961039	0.032241953
HSA04912:GNRH SIGNALING PATHWAY	PRKCB, ADCY2, ITPR3, CACNA1C	14.39874411	0.032241953
HSA04972:PANCREATIC SECRETION	CTRL, PRKCB, ADCY2, ITPR3	14.0890937	0.032241953
HSA04713:CIRCADIAN ENTRAINMENT	PRKCB, ADCY2, ITPR3, CACNA1C	13.7924812	0.032241953
HSA04723:RETROGRADE ENDOCANNABINOID SIGNALING	PRKCB, ADCY2, ITPR3, CACNA1C	12.97312588	0.033590835
HSA04725:CHOLINERGIC SYNAPSE	PRKCB, ADCY2, ITPR3, CACNA1C	11.8043758	0.037018917
HSA04724:GLUTAMATERGIC SYNAPSE	PRKCB, ADCY2, ITPR3, CACNA1C	11.49373434	0.037018917
HSA04270:VASCULAR SMOOTH MUSCLE CONTRACTION	PRKCB, ADCY2, ITPR3, CACNA1C	11.1990232	0.037018917
HSA04022:CGMP-PKG SIGNALING PATHWAY	NFATC3, ADCY2, ITPR3, CACNA1C	8.292947559	0.077981143
HSA04020:CALCIUM SIGNALING PATHWAY	PRKCB, ADCY2, ITPR3, CACNA1C	7.320031923	0.100904234
HSA04720:LONG-TERM POTENTIATION	PRKCB, ITPR3, CACNA1C	14.88961039	0.101277547
HSA04918:THYROID HORMONE SYNTHESIS	PRKCB, ADCY2, ITPR3	14.03877551	0.105688738
HSA04971:GASTRIC ACID SECRETION	PRKCB, ADCY2, ITPR3	13.46183953	0.107263228
HSA04727:GABAERGIC SYNAPSE	PRKCB, ADCY2, CACNA1C	11.56134454	0.129654661
HSA04970:SALIVARY SECRETION	PRKCB, ADCY2, ITPR3	11.4269103	0.129654661
HSA04750:INFLAMMATORY MEDIATOR REGULATION OF TRP CHANNELS	PRKCB, ADCY2, ITPR3	10.02769679	0.14862936

SUPPLEMENTARY TABLE 3.T2D PGS GENE ENRICHMENT

KEGG	Genes	Fold Enrichment	FDR
HSA04921:OXYTOCIN SIGNALING PATHWAY	CACNB2, NFATC3, ITPR3, CACNA2D4, CACNA1C, ADCY1	10.58307692	0.016533875
HSA05414:DILATED CARDIOMYOPATHY	CACNB2, CACNA2D4, CACNA1C, ADCY1	12.5989011	0.110122319
HSA04911:INSULIN SECRETION	PCLO, ITPR3, CACNA1C, ADCY1	12.45067873	0.110122319
HSA04010:MAPK SIGNALING PATHWAY	CACNB2, DAXX, NFATC3, CACNA2D4, CACNA1C	5.228792946	0.252582164
HSA04261:ADRENERGIC SIGNALING IN CARDIOMYOCYTES	CACNB2, CACNA2D4, CACNA1C, ADCY1	7.668896321	0.252582164
HSA04022:CGMP-PKG SIGNALING PATHWAY	NFATC3, ITPR3, CACNA1C, ADCY1	6.698149951	0.275895042
HSA04720:LONG-TERM POTENTIATION	ITPR3, CACNA1C, ADCY1	12.02622378	0.275895042
HSA05412:ARRHYTHMOGENIC RIGHT VENTRICULAR CARDIOMYOPATHY (ARVC)	CACNB2, CACNA2D4, CACNA1C	11.8467279	0.275895042
HSA04920:ADIPOCYTOKINE SIGNALING PATHWAY	TRADD, AGRP, ADIPOR2	11.33901099	0.275895042
HSA04260:CARDIAC MUSCLE CONTRACTION	CACNB2, CACNA2D4, CACNA1C	10.58307692	0.275895042
HSA05410:HYPERTROPHIC CARDIOMYOPATHY (HCM)	CACNB2, CACNA2D4, CACNA1C	10.1760355	0.275895042
HSA04925:ALDOSTERONE SYNTHESIS AND SECRETION	ITPR3, CACNA1C, ADCY1	9.799145299	0.275895042
HSA04912:GNRH SIGNALING PATHWAY	ITPR3, CACNA1C, ADCY1	8.722316145	0.294950189
HSA04972:PANCREATIC SECRETION	CTRL, ITPR3, ADCY1	8.534739454	0.294950189
HSA04713:CIRCADIAN ENTRAINMENT	ITPR3, CACNA1C, ADCY1	8.355060729	0.294950189
HSA04723:RETROGRADE ENDOCANNABINOID SIGNALING	ITPR3, CACNA1C, ADCY1	7.858720487	0.308655949
HSA04725:CHOLINERGIC SYNAPSE	ITPR3, CACNA1C, ADCY1	7.150727651	0.337253376
HSA04724:GLUTAMATERGIC SYNAPSE	ITPR3, CACNA1C, ADCY1	6.962550607	0.337253376
HSA04270:VASCULAR SMOOTH MUSCLE CONTRACTION	ITPR3, CACNA1C, ADCY1	6.784023669	0.337253376

Supplementary Table 4. Correlation matrix of continuous variables

Variables	Waist to hip ratio	BMI	BMI impedance	Trunk fat percentage	Whole-body fat percentage	Grip strength	Total food intake	Total energy intake	Sugar intake
Waist to hip ratio	1	0.451368034	0.451290232	0.396862204	-0.134205189	0.4325529	0.083145509	0.162972791	0.034015781
BMI	0.451368034	1	0.999941824	0.869580833	0.561798562	0.069927756	0.048753396	0.01926263	-0.036533145
BMI impedance	0.451290232	0.999941824	1	0.869505376	0.561831311	0.06993537	0.048731474	0.019213758	-0.036564443
Trunk fat percentage	0.396862204	0.869580833	0.869505376	1	0.694492208	0.00582614	0.031160448	-0.005960375	-0.052434013
Whole-body fat percentage	-0.134205189	0.561798562	0.561831311	0.694492208	1	-0.536431266	-0.078677266	-0.196434621	-0.115135907
Grip strength	0.4325529	0.069927756	0.06993537	0.00582614	-0.536431266	1	0.112805966	0.21257189	0.07286673
Total food intake	0.083145509	0.048753396	0.048731474	0.031160448	-0.078677266	0.112805966	1	0.546950231	0.48763336
Total energy intake	0.162972791	0.01926263	0.019213758	-0.005960375	-0.196434621	0.21257189	0.546950231	1	0.683616697
Sugar intake	0.034015781	-0.036533145	-0.036564443	-0.052434013	-0.115135907	0.07286673	0.48763336	0.683616697	1

Supplementary Table 5. Linear PRS Models Results for the continuous phenotype clusters' PCs

Variable	PRS	Method	Estimate	Lower CI bound	Upper CI bound	R^2	p adj.
Body fat PC	BD	PRS	0.00201	-8.08E-04	0.004828	0.020421	0.257441
Body fat PC	BD	PC1	0.001729	-0.00108	0.00454	0.02042	0.324343
Body fat PC	BD	PC2	-0.00348	-0.00631	-6.59E-04	0.020429	0.05282
Body fat PC	SCZ	PRS	-0.00207	-0.00494	7.92E-04	0.020422	0.257441
Body fat PC	SCZ	PC1	-0.00105	-0.00389	0.001782	0.020418	0.576233
Body fat PC	SCZ	PC2	0.004501	0.001679	0.007324	0.020437	0.009426
Body fat PC	T2D	PRS	0.007627	0.00466	0.010595	0.020469	6.39E-06
Body fat PC	T2D	PC1	0.007598	0.004677	0.010518	0.020471	6.39E-06
Body fat PC	T2D	PC2	0.003553	7.22E-04	0.006385	0.02043	0.05282
Diet PC	BD	PRS	0.005065	8.11E-04	0.009318	0.033318	0.058865
Diet PC	BD	PC1	0.004038	-2.08E-04	0.008285	0.033309	0.168262
Diet PC	BD	PC2	-0.00379	-0.00805	4.74E-04	0.033306	0.200058
Diet PC	SCZ	PRS	5.85E-04	-0.00372	0.004893	0.033293	0.888863
Diet PC	SCZ	PC1	4.88E-04	-0.00379	0.004764	0.033293	0.889031
Diet PC	SCZ	PC2	0.002718	-0.00154	0.006977	0.0333	0.316379
Diet PC	T2D	PRS	-0.0015	-0.00594	0.002938	0.033294	0.596345
Diet PC	T2D	PC1	-0.00196	-0.00634	0.002415	0.033296	0.512347
Diet PC	T2D	PC2	0.003095	-0.00117	0.007366	0.033302	0.257441
WHR/Grip PC	BD	PRS	5.92E-04	-0.00101	0.002198	0.67822	0.576233
WHR/Grip PC	BD	PC1	0.00121	-3.91E-04	0.002812	0.678221	0.257441
WHR/Grip PC	BD	PC2	-9.61E-06	-0.00162	0.0016	0.67822	0.990661
WHR/Grip PC	SCZ	PRS	1.33E-04	-0.0015	0.001764	0.67822	0.906947
WHR/Grip PC	SCZ	PC1	0.001172	-4.44E-04	0.002787	0.678221	0.257441
WHR/Grip PC	SCZ	PC2	0.002524	9.16E-04	0.004131	0.678226	0.009426
WHR/Grip PC	T2D	PRS	0.002726	0.001035	0.004416	0.678226	0.009426
WHR/Grip PC	T2D	PC1	0.001439	-2.25E-04	0.003102	0.678222	0.202566
WHR/Grip PC	T2D	PC2	0.002622	0.001009	0.004235	0.678226	0.009426

Supplementary Table 6. Linear PRS Models Results

Variable	PRS	Estimate	Lower CI bound	Upper CI bound	R^2	p adj.
BMI	BP	0.001787	-0.00102	0.004593	0.012991	0.312292
BMI	SCZ	-0.0025	-0.00535	0.000355	0.012994	0.174482
BMI	T2D	0.008531	0.005575	0.011487	0.013053	2.22E-07
BMI (imp)	BP	0.002035	-0.00079	0.004862	0.012889	0.278493
BMI (imp)	SCZ	-0.00221	-0.00508	0.000662	0.012889	0.242155
BMI (imp)	T2D	0.008576	0.005599	0.011553	0.01295	2.22E-07
Total caloric intake	BP	0.003293	-0.00089	0.00748	0.063395	0.232165
Total caloric intake	SCZ	-0.00146	-0.0057	0.002783	0.063386	0.58763
Total caloric intake	T2D	-0.00383	-0.00819	0.000542	0.063398	0.174482
Food weight	BP	0.007318	0.003024	0.011613	0.014744	0.003772
Food weight	SCZ	0.002888	-0.00146	0.007237	0.014699	0.30496
Food weight	T2D	-0.003	-0.00747	0.001484	0.014699	0.30496
Grip strength	BP	0.000597	-0.00125	0.002445	0.573229	0.600351
Grip strength	SCZ	-0.00408	-0.00596	-0.0022	0.573245	0.000138
Grip strength	T2D	-0.00426	-0.00621	-0.00232	0.573245	0.00013
Sugar intake	BP	0.00256	-0.00174	0.006861	0.011606	0.352106
Sugar intake	SCZ	0.000319	-0.00404	0.004675	0.0116	0.896884
Sugar intake	T2D	0.002936	-0.00155	0.007422	0.011607	0.30496
Trunk fat %	BP	0.002248	-0.00058	0.005075	0.012558	0.23002
Trunk fat %	SCZ	-0.00294	-0.00581	-6.56E-05	0.012561	0.105813
Trunk fat %	T2D	0.005008	0.00203	0.007987	0.012575	0.004187
Waist-to-hip ratio	BP	0.00035	-0.00175	0.002452	0.445167	0.763247
Waist-to-hip ratio	SCZ	0.004284	0.002147	0.006421	0.445184	0.000494
Waist-to-hip ratio	T2D	0.008892	0.006677	0.011107	0.445237	2.88E-13
Whole-body fat %	BP	0.001091	-0.00102	0.0032	0.451015	0.426151
Whole-body fat %	SCZ	0.000827	-0.00132	0.00297	0.451015	0.543078
Whole-body fat %	T2D	0.005275	0.003054	0.007496	0.451039	2.91E-05

Supplementary Table 7. Linear PC1 Model Results

Variable	PRS	Estimate	Lower CI bound	Upper CI bound	R^2	p adj.
BMI	BD	0.001451	-0.00135	0.004252	0.01299	0.426151
BMI	SCZ	-0.00128	-0.00411	0.001545	0.012989	0.482961
BMI	T2D	0.007501	0.004593	0.010409	0.01304	4.98E-06
BMI (imp)	BD	0.001804	-0.00102	0.004624	0.012888	0.312292
BMI (imp)	SCZ	-0.00104	-0.00389	0.001804	0.012886	0.56385
BMI (imp)	T2D	0.007475	0.004546	0.010404	0.012936	5.75E-06
Total caloric intake	BD	0.00187	-0.00231	0.006049	0.063388	0.482961
Total caloric intake	SCZ	-0.00198	-0.00618	0.002233	0.063388	0.474831
Total caloric intake	T2D	-0.00607	-0.01038	-0.00176	0.063419	0.018684
Food weight	BD	0.007828	0.003542	0.012115	0.014752	0.001861
Food weight	SCZ	0.00355	-0.00077	0.007867	0.014703	0.211446
Food weight	T2D	-0.00119	-0.00561	0.003234	0.014692	0.664609
Grip strength	BD	0.001299	-0.00054	0.003143	0.57323	0.288457
Grip strength	SCZ	-0.00288	-0.00474	-0.00102	0.573237	0.008936
Grip strength	T2D	-0.00597	-0.00788	-0.00405	0.573262	2.08E-08
Sugar intake	BD	0.000997	-0.0033	0.005291	0.0116	0.682762
Sugar intake	SCZ	4.49E-06	-0.00432	0.004328	0.011599	0.998376
Sugar intake	T2D	0.002433	-0.00199	0.006859	0.011605	0.399842
Trunk fat %	BD	0.00206	-0.00076	0.004882	0.012557	0.274237
Trunk fat %	SCZ	-0.00194	-0.00479	0.000903	0.012557	0.299011
Trunk fat %	T2D	0.005909	0.002979	0.00884	0.012585	0.000482
Waist-to-hip ratio	BD	0.000709	-0.00139	0.002807	0.445167	0.58767
Waist-to-hip ratio	SCZ	0.004843	0.002726	0.006959	0.445189	5.92E-05
Waist-to-hip ratio	T2D	0.008489	0.00631	0.010667	0.445233	9.06E-13
Whole-body fat %	BD	0.000939	-0.00116	0.003043	0.451015	0.482961
Whole-body fat %	SCZ	0.001063	-0.00106	0.003185	0.451015	0.440571
Whole-body fat %	T2D	0.006984	0.004799	0.009169	0.451059	1.01E-08

Supplementary Table 8. Linear PC2 Models Results

Variable	PRS	Estimate	Lower CI bound	Upper CI bound	R^2	p adj.
BMI	BD	-0.00378	-0.00659	-0.00096	0.013002	0.026461
BMI	SCZ	0.004941	0.00213	0.007752	0.013012	0.002891
BMI	T2D	0.004583	0.001763	0.007402	0.013008	0.005861
BMI (imp)	BD	-0.0037	-0.00654	-0.00087	0.012898	0.031423
BMI (imp)	SCZ	0.004929	0.002098	0.00776	0.012909	0.003069
BMI (imp)	T2D	0.004491	0.001651	0.007331	0.012905	0.007478
Total caloric intake	BD	-0.00292	-0.00711	0.001278	0.063393	0.291845
Total caloric intake	SCZ	0.000919	-0.00327	0.005111	0.063385	0.693162
Total caloric intake	T2D	0.00447	0.000266	0.008673	0.063404	0.094029
Food weight	BD	-0.00175	-0.00605	0.002549	0.014694	0.521189
Food weight	SCZ	0.004449	0.00015	0.008749	0.01471	0.104427
Food weight	T2D	-0.00104	-0.00535	0.003274	0.014692	0.680641
Grip strength	BD	0.000509	-0.00134	0.002362	0.573229	0.664283
Grip strength	SCZ	0.002812	0.000961	0.004663	0.573237	0.010233
Grip strength	T2D	0.002046	0.000189	0.003903	0.573233	0.08313
Sugar intake	BD	-0.00485	-0.00916	-0.00054	0.011623	0.078936
Sugar intake	SCZ	0.001771	-0.00254	0.006077	0.011603	0.521189
Sugar intake	T2D	0.004074	-0.00024	0.008392	0.011616	0.141095
Trunk fat %	BD	-0.00303	-0.00586	-0.00019	0.012562	0.094029
Trunk fat %	SCZ	0.004229	0.001397	0.007062	0.012571	0.011569
Trunk fat %	T2D	0.002693	-0.00015	0.005534	0.01256	0.141095
Waist-to-hip ratio	BD	-0.00054	-0.00265	0.001566	0.445167	0.672443
Waist-to-hip ratio	SCZ	0.001388	-0.00072	0.003494	0.445168	0.30496
Waist-to-hip ratio	T2D	0.002364	0.000252	0.004477	0.445172	0.078936
Whole-body fat %	BD	-0.00191	-0.00403	0.0002	0.451018	0.161953
Whole-body fat %	SCZ	0.002153	4.09E-05	0.004265	0.451019	0.105813
Whole-body fat %	T2D	0.000508	-0.00161	0.002626	0.451014	0.680641

Supplementary Table 9. Interactions BMI ~ BD PGS$_{oxt}$ PC2

Term	Estimate	Error	P value
(Intercept)	0.010712	0.005987	0.073559
PGS	-0.00513	0.004149	0.216296
BMI_imp	0.999211	1.92E-04	0
trunk_fat	0.002461	3.16E-04	6.27E-15
wb_fat_perc	-7.45E-04	1.38E-04	7.26E-08
genetic_sexmale	-0.00564	6.05E-04	1.15E-20
age	1.63E-04	1.57E-04	0.301081
age_sq	-1.10E-06	1.42E-06	0.436406
PC1	-0.00909	0.086491	0.916334
PC2	-0.06062	0.061253	0.32237
PC3	0.024401	0.06132	0.690681
PC4	0.029699	0.062219	0.633131
PC5	-0.06013	0.061363	0.32717
PC6	0.004955	0.061254	0.935531
PC7	-0.0362	0.061288	0.554789
PC8	-0.05734	0.06137	0.350167
PC9	-0.02823	0.061214	0.644673
PC10	-0.05195	0.061989	0.402042
PGS:BMI_imp	3.03E-04	1.88E-04	0.107916
PGS:trunk_fat	2.12E-04	2.71E-04	0.43383
BMI_imp:trunk_fat	-4.01E-05	9.60E-06	2.98E-05
PGS:wb_fat_perc	9.36E-05	1.27E-04	0.462783
BMI_imp:wb_fat_perc	1.14E-05	5.47E-06	0.036594
trunk_fat:wb_fat_perc	-2.44E-05	6.72E-06	2.85E-04
PGS:BMI_imp:trunk_fat	-1.59E-05	9.06E-06	0.078765
PGS:BMI_imp:wb_fat_perc	-7.69E-06	5.36E-06	0.150955
PGS:trunk_fat:wb_fat_perc	3.88E-06	6.53E-06	0.552545
BMI_imp:trunk_fat:wb_fat_perc	5.54E-07	2.12E-07	0.009014
PGS:BMI_imp:trunk_fat:wb_fat_perc	1.68E-07	2.09E-07	0.421179

Supplementary Table 10. Interactions BMI (impedance) ~ BD PGS$_{oxt}$ PC2

Term	Estimate	Error	P value
(Intercept)	0.00339	0.005989	0.571419
PGS	0.003663	0.004151	0.377577
BMI	0.999946	1.92E-04	0
trunk_fat	-0.00218	3.16E-04	5.33E-12
wb_fat_perc	7.66E-04	1.38E-04	3.12E-08
genetic_sexmale	0.007962	6.05E-04	1.67E-39
age	-1.63E-04	1.57E-04	0.300358
age_sq	9.25E-07	1.42E-06	0.514707
PC1	0.009199	0.086522	0.915326
PC2	0.059442	0.061275	0.331996
PC3	-0.01626	0.061342	0.790981
PC4	-0.06278	0.062241	0.313131
PC5	0.072999	0.061385	0.234356
PC6	-0.01086	0.061276	0.859366
PC7	0.041148	0.06131	0.50213
PC8	0.062868	0.061392	0.305816
PC9	0.024487	0.061235	0.689244
PC10	0.054566	0.062011	0.378898
PGS:BMI	-1.97E-04	1.89E-04	0.297027
PGS:trunk_fat	-3.46E-05	2.71E-04	0.898551
BMI:trunk_fat	3.89E-05	9.61E-06	5.02E-05
PGS:wb_fat_perc	-6.33E-05	1.28E-04	0.619715
BMI:wb_fat_perc	-8.77E-06	5.47E-06	0.108902
trunk_fat:wb_fat_perc	2.70E-05	6.72E-06	5.89E-05
PGS:BMI:trunk_fat	6.07E-06	9.07E-06	0.502876
PGS:BMI:wb_fat_perc	2.79E-06	5.36E-06	0.602587
PGS:trunk_fat:wb_fat_perc	1.07E-06	6.53E-06	0.869384
BMI:trunk_fat:wb_fat_perc	-4.89E-07	2.12E-07	0.021032
PGS:BMI:trunk_fat:wb_fat_perc	-8.57E-08	2.09E-07	0.681877

Supplementary Table 11. Interactions Sugar intake ~ BD PC2 PGS_{oxt}

Term	Estimate	Error	P value
(Intercept)	10.60051629	4.074529618	0.009278
PGS	-4.777683706	0.689560693	4.26E-12
mean_energy_intake	0.010745435	7.08E-05	0
mean_food_intake	0.007635509	2.03E-04	0.00E+00
genetic_sexmale	-8.681270566	0.170026473	0
age	-0.961184703	0.148284945	9.07E-11
age_sq	0.012566598	0.001346608	1.05E-20
PC1	31.5262189	80.48419813	0.695275
PC2	-189.805714	57.28198556	9.21E-04
PC3	615.1388853	58.00328923	2.86E-26
PC4	-1617.484939	64.55058326	2.32E-138
PC5	607.0822713	57.92710436	1.08E-25
PC6	44.42449593	57.03767305	0.436062
PC7	190.1783133	57.38114575	9.19E-04
PC8	208.6407481	58.11318193	3.30E-04
PC9	-189.5736136	57.06389085	8.93E-04
PC10	50.06811263	61.58145304	0.416197
PGS:mean_energy_intake	6.74E-04	7.28E-05	2.04E-20
PGS:mean_food_intake	0.001530698	2.09E-04	2.23E-13
mean_energy_intake:mean_food_intake	2.96E-07	1.65E-08	2.60E-71
PGS:mean_energy_intake:mean_food_intake	-2.11E-07	1.73E-08	3.28E-34

Supplementary Table 12. Interactions Food weight ~ BD PGS$_{oxt}$

Term	Estimate	Error	P value
(Intercept)	1000.251723	80.51525289	2.02E-35
PGS	55827.81811	9320.913464	2.11E-09
mean_energy_intake	0.123558966	0.004532132	2.28E-163
mean_sugar_intake	3.149673964	0.27655127	4.84E-30
genetic_sexmale	-12.00824531	3.04865475	8.19E-05
age	30.18786678	2.637663851	2.55E-30
age_sq	-0.298542431	0.023954733	1.23E-35
PC1	-1642.48523	1431.970857	0.251379
PC2	1576.369583	1019.191965	0.121941
PC3	-2894.794039	1031.846799	0.005025
PC4	14573.67081	1148.360358	6.86E-37
PC5	-6196.167036	1030.244146	1.81E-09
PC6	262.1316149	1014.721035	0.796153
PC7	-2075.872331	1020.772264	0.04199
PC8	-2615.864634	1033.237117	0.011351
PC9	3647.330341	1015.25806	3.28E-04
PC10	-2347.918999	1094.64908	0.031962
PGS:mean_energy_intake	-4.252906152	1.13215778	1.72E-04
PGS:mean_sugar_intake	-485.1013989	69.56443596	3.10E-12
mean_energy_intake:mean_sugar_intake	1.07E-05	1.73E-05	0.535918
PGS:mean_energy_intake:mean_sugar_intake	0.039377598	0.0043897	2.98E-19

Supplementary Table 13. Interactions Food weight ~ BD PC PGS_{oxt}

Term	Estimate	Error	P value
(Intercept)	782.4504786	72.02633385	1.75E-27
PGS	34.25823612	7.174569752	1.80E-06
mean_energy_intake	0.140257791	9.37E-04	0
mean_sugar_intake	5.064372084	0.056457372	0
genetic_sexmale	-11.96391141	3.048688273	8.70E-05
age	30.11197922	2.637675447	3.55E-30
age_sq	-0.297875944	0.023954852	1.74E-35
PC1	-1666.13017	1431.991612	0.244626
PC2	1596.124364	1019.20494	0.117339
PC3	-2891.923636	1031.854398	0.005069
PC4	14610.13443	1147.901247	4.28E-37
PC5	-6178.084301	1030.29883	2.02E-09
PC6	215.1756049	1014.694133	0.832061
PC7	-2043.170217	1020.741127	0.045323
PC8	-2529.53844	1033.097705	0.014346
PC9	3648.791913	1015.279643	3.26E-04
PC10	-2186.068607	1093.245136	0.045543
PGS:mean_energy_intake	-0.001706548	8.82E-04	0.053071
PGS:mean_sugar_intake	-0.355085594	0.053439912	3.05E-11
mean_energy_intake:mean_sugar_intake	-1.44E-04	3.53E-06	0
PGS:mean_energy_intake:mean_sugar_intake	2.51E-05	3.16E-06	2.11E-15

Supplementary Table 14. Logistic Regression PRS Models Results

Variables	PRS	Odds ratio	Lower CI bound	Upper CI bound	Tjur's pseudo R2	p adj.
Able to confide	BD	0.989001	0.981933	0.99612	0.013641	0.022577
Able to confide	SCZ	1.005416	0.998118	1.012768	0.013627	0.40477
Able to confide	T2D	1.014083	1.006456	1.021769	0.013652	0.003395
Loneliness	BD	1.002767	0.995401	1.010189	0.010204	0.616808
Loneliness	SCZ	1.018163	1.010567	1.025816	0.010253	8.87E-05
Loneliness	T2D	1.008575	1.000803	1.016408	0.010214	0.137349
Social activity	BD	1.008512	1.002333	1.014728	0.002879	0.035337
Social activity	SCZ	1.002403	0.996158	1.008686	0.002865	0.616808
Social activity	T2D	0.996568	0.990131	1.003047	0.002866	0.469069
Social contact	BD	1.001155	0.984386	1.01821	0.006757	0.905579
Social contact	SCZ	1.00125	0.984155	1.018642	0.006757	0.905579
Social contact	T2D	0.98922	0.971618	1.007141	0.00676	0.469069

Supplementary Table 15. Logistic Regression PC1 Models Results

Variables	PRS	Odds ratio	Lower CI bound	Upper CI bound	Tjur's pseudo R2	p adj.
Able to confide	BD	0.990013	0.982956	0.997122	0.013637	0.035337
Able to confide	SCZ	1.002329	0.995124	1.009586	0.013624	0.647215
Able to confide	T2D	1.005568	0.998128	1.013064	0.013627	0.40477
Loneliness	BD	1.000445	0.993113	1.007832	0.010203	0.905579
Loneliness	SCZ	1.015952	1.008443	1.023517	0.010241	0.000522
Loneliness	T2D	1.007158	0.999516	1.014857	0.010211	0.239168
Social activity	BD	1.0087	1.002533	1.014905	0.002879	0.035337
Social activity	SCZ	1.003279	0.997091	1.009506	0.002866	0.469069
Social activity	T2D	0.99575	0.989423	1.002116	0.002867	0.469069
Social contact	BD	1.00105	0.98431	1.018076	0.006757	0.905579
Social contact	SCZ	1.004602	0.987623	1.021873	0.006757	0.672251
Social contact	T2D	0.983133	0.965912	1.000661	0.006767	0.236795

Supplementary Table 16. Logistic Regression PC2 Models Results

Variables	PRS	Odds ratio	Lower CI bound	Upper CI bound	Tjur's pseudo R2	p adj.
Able to confide	BD	1.003915	0.996726	1.011157	0.013626	0.469069
Able to confide	SCZ	0.997293	0.990159	1.004478	0.013624	0.616808
Able to confide	T2D	1.002417	0.995227	1.009659	0.013624	0.647215
Loneliness	BD	0.995464	0.988138	1.002845	0.010206	0.469069
Loneliness	SCZ	0.993769	0.986465	1.001128	0.01021	0.316899
Loneliness	T2D	0.997871	0.990521	1.005275	0.010204	0.664306
Social activity	BD	0.996089	0.989968	1.002248	0.002867	0.469069
Social activity	SCZ	1.002438	0.996283	1.00863	0.002865	0.616808
Social activity	T2D	0.99657	0.990431	1.002747	0.002866	0.469069
Social contact	BD	0.99469	0.977923	1.011745	0.006758	0.647215
Social contact	SCZ	1.01024	0.993247	1.027524	0.006758	0.469069
Social contact	T2D	1.009892	0.992804	1.027275	0.006761	0.469069

Supplementary Table 17. Interactions Ability to confide ~ BD PGS_{oxt}

Term	Estimate	Error	P value
(Intercept)	-2.71151	0.186504	6.90E-48
PGS	-0.01574	0.007845	0.044761601
loneliness1	1.082437	0.01479	0
family_friends1	0.0689	0.053384	0.196825951
social_activity1	-0.02372	0.089353	0.790620735
social_activity0	-0.20979	0.009488	2.55E-108
genetic_sexmale	0.585465	0.007596	0
age	0.015443	0.006797	0.02307895
age_sq	2.87E-05	6.11E-05	0.63898299
PC1	-3.61692	3.697183	0.327931007
PC2	-1.06564	2.610583	0.683126501
PC3	16.18298	2.605831	5.29E-10
PC4	-58.9502	2.49629	2.69E-123
PC5	17.97558	2.599293	4.66E-12
PC6	1.564015	2.620649	0.55063803
PC7	4.67161	2.620781	0.074663547
PC8	2.606803	2.613001	0.318459893
PC9	-4.14473	2.617677	0.113338908
PC10	-16.2057	2.63532	7.78E-10
PGS:loneliness1	0.031239	0.014727	0.033907113
PGS:family_friends1	0.063284	0.052777	0.23049444
loneliness1:family_friends1	-0.47322	0.082498	9.69E-09
PGS:social_activity1	-0.07815	0.090826	0.389528334
PGS:social_activity0	0.007484	0.009503	0.430984511
loneliness1:social_activity1	-0.28073	0.159658	0.078692758
loneliness1:social_activity0	-0.083	0.018246	5.39E-06
family_friends1:social_activity1	-0.65589	0.742457	0.377016147
family_friends1:social_activity0	-0.09419	0.063198	0.136119909
PGS:loneliness1:family_friends1	-0.06902	0.082274	0.401521787
PGS:loneliness1:social_activity0	-0.04365	0.018249	0.016762001

PGS:family_friends1:social_activity1	-0.70213	0.590739	0.234608835
PGS:family_friends1:social_activity0	-0.12576	0.062448	0.044020644
loneliness1:family_friends1:social_activity1	1.835671	0.976684	0.060177169
loneliness1:family_friends1:social_activity0	0.018611	0.100238	0.85270462
PGS:loneliness1:family_friends1:social_activity1	-0.20742	0.975746	0.831657643
PGS:loneliness1:family_friends1:social_activity0	0.139847	0.099967	0.161835615

Supplementary Table 18. Interactions Ability to confide ~ BD PC PGS$_{oxt}$

Term	Estimate	Error	P value
(Intercept)	-2.71151	0.186504	6.90E-48
PGS	-0.01574	0.007845	0.044762
loneliness1	1.082437	0.01479	0
family_friends1	0.0689	0.053384	0.196826
social_activity1	-0.02372	0.089353	0.790621
social_activity0	-0.20979	0.009488	2.55E-108
genetic_sexmale	0.585465	0.007596	0
age	0.015443	0.006797	0.023079
age_sq	2.87E-05	6.11E-05	0.638983
PC1	-3.61692	3.697183	0.327931
PC2	-1.06564	2.610583	0.683127
PC3	16.18298	2.605831	5.29E-10
PC4	-58.9502	2.49629	2.69E-123
PC5	17.97558	2.599293	4.66E-12
PC6	1.564015	2.620649	0.550638
PC7	4.67161	2.620781	0.074664
PC8	2.606803	2.613001	0.31846
PC9	-4.14473	2.617677	0.113339
PC10	-16.2057	2.63532	7.78E-10
PGS:loneliness1	0.031239	0.014727	0.033907
PGS:family_friends1	0.063284	0.052777	0.230494
loneliness1:family_friends1	-0.47322	0.082498	9.69E-09
PGS:social_activity1	-0.07815	0.090826	0.389528
PGS:social_activity0	0.007484	0.009503	0.430985
loneliness1:social_activity1	-0.28073	0.159658	0.078693
loneliness1:social_activity0	-0.083	0.018246	5.39E-06
family_friends1:social_activity1	-0.65589	0.742457	0.377016
family_friends1:social_activity0	-0.09419	0.063198	0.13612
PGS:loneliness1:family_friends1	-0.06902	0.082274	0.401522

PGS:loneliness1:social_activity0	-0.04365	0.018249	0.016762
PGS:family_friends1:social_activity1	-0.70213	0.590739	0.234609
PGS:family_friends1:social_activity0	-0.12576	0.062448	0.044021
loneliness1:family_friends1:social_activity1	1.835671	0.976684	0.060177
loneliness1:family_friends1:social_activity0	0.018611	0.100238	0.852705
PGS:loneliness1:family_friends1:social_activity1	-0.20742	0.975746	0.831658
PGS:loneliness1:family_friends1:social_activity0	0.139847	0.099967	0.161836

Supplementary Table 19. Interactions BMI ~ SCZ PGS_{oxt} PC2

Term	Estimate	Error	P value
(Intercept)	0.010708	0.005986	0.073634
PGS	9.11E-04	0.004195	0.828156
BMI_imp	0.999209	1.92E-04	0
trunk_fat	0.002479	3.16E-04	3.96E-15
wb_fat_perc	-7.46E-04	1.38E-04	6.97E-08
genetic_sexmale	-0.00564	6.05E-04	1.09E-20
age	1.63E-04	1.57E-04	0.299817
age_sq	-1.11E-06	1.42E-06	0.435071
PC1	-0.00936	0.086492	0.913855
PC2	-0.06247	0.061253	0.307765
PC3	0.027267	0.061315	0.656538
PC4	0.021387	0.062352	0.731596
PC5	-0.05599	0.06135	0.361473
PC6	0.004218	0.061247	0.945094
PC7	-0.03469	0.061277	0.571305
PC8	-0.05327	0.061325	0.385001
PC9	-0.02848	0.061213	0.641695
PC10	-0.04793	0.061853	0.438442
PGS:BMI_imp	-2.44E-05	1.92E-04	0.89888
PGS:trunk_fat	-3.68E-04	2.70E-04	0.17235
BMI_imp:trunk_fat	-4.05E-05	9.60E-06	2.40E-05
PGS:wb_fat_perc	1.14E-04	1.30E-04	0.380214
BMI_imp:wb_fat_perc	1.15E-05	5.47E-06	0.034716
trunk_fat:wb_fat_perc	-2.49E-05	6.72E-06	2.06E-04
PGS:BMI_imp:trunk_fat	1.36E-05	9.00E-06	0.131291
PGS:BMI_imp:wb_fat_perc	-4.65E-06	5.51E-06	0.3983
PGS:trunk_fat:wb_fat_perc	-3.10E-07	6.49E-06	0.961912
BMI_imp:trunk_fat:wb_fat_perc	5.67E-07	2.12E-07	0.007506
PGS:BMI_imp:trunk_fat:wb_fat_perc	6.39E-09	2.09E-07	0.975582

Supplementary Table 20. Interactions BMI (impedance) ~ SCZ PGS_{oxt} PC2

Term	Estimate	Error	P value
(Intercept)	0.003302	0.005988	0.581352
PGS	-0.00306	0.004197	0.465283
BMI	0.999947	1.92E-04	0
trunk_fat	-0.00217	3.16E-04	5.76E-12
wb_fat_perc	7.67E-04	1.38E-04	3.02E-08
genetic_sexmale	0.007956	6.05E-04	1.90E-39
age	-1.61E-04	1.57E-04	0.305443
age_sq	9.10E-07	1.42E-06	0.521538
PC1	0.009136	0.086522	0.915909
PC2	0.059627	0.061274	0.330495
PC3	-0.01703	0.061336	0.781283
PC4	-0.06235	0.062373	0.317526
PC5	0.072318	0.061371	0.238648
PC6	-0.01092	0.061268	0.858501
PC7	0.04096	0.061298	0.503995
PC8	0.062179	0.061346	0.310784
PC9	0.024682	0.061233	0.686888
PC10	0.053491	0.061875	0.387313
PGS:BMI	1.03E-04	1.92E-04	0.589889
PGS:trunk_fat	3.12E-05	2.70E-04	0.907944
BMI:trunk_fat	3.89E-05	9.60E-06	5.17E-05
PGS:wb_fat_perc	6.89E-05	1.30E-04	0.595873
BMI:wb_fat_perc	-8.81E-06	5.47E-06	0.107318
trunk_fat:wb_fat_perc	2.69E-05	6.72E-06	6.08E-05
PGS:BMI:trunk_fat	-2.26E-06	9.00E-06	0.802048
PGS:BMI:wb_fat_perc	-8.31E-07	5.51E-06	0.880172
PGS:trunk_fat:wb_fat_perc	-1.40E-06	6.50E-06	0.829711
BMI:trunk_fat:wb_fat_perc	-4.88E-07	2.12E-07	0.021308
PGS:BMI:trunk_fat:wb_fat_perc	-6.26E-09	2.09E-07	0.976078

Supplementary Table 21. Interactions Trunk fat % ~ SCZ PGS$_{oxt}$ PC2

Term	Estimate	Error	P value
(Intercept)	20.038	0.204343	0
PGS	0.040025	0.168862	0.812636
BMI	-0.82993	0.15658	1.16E-07
BMI_imp	-1.54643	0.156469	4.94E-23
wb_fat_perc	0.024453	0.004449	3.89E-08
genetic_sexmale	6.998882	0.009614	0
age	-0.00393	0.003923	0.315884
age_sq	-2.33E-04	3.54E-05	4.35E-11
PC1	0.966744	2.158401	0.654227
PC2	7.56372	1.528517	7.48E-07
PC3	-3.192	1.530053	0.036961
PC4	67.08168	1.552255	0
PC5	-11.6628	1.530841	2.57E-14
PC6	-8.42679	1.528359	3.52E-08
PC7	11.14967	1.529027	3.06E-13
PC8	22.95108	1.529827	7.27E-51
PC9	2.719701	1.527537	0.075003
PC10	86.44164	1.536828	0
PGS:BMI	-0.03261	0.159815	0.838295
PGS:BMI_imp	0.035738	0.159796	0.823033
BMI:BMI_imp	0.048937	2.34E-04	0
PGS:wb_fat_perc	-0.00993	0.004414	0.024544
BMI:wb_fat_perc	0.02446	0.004291	1.19E-08
BMI_imp:wb_fat_perc	0.021017	0.004287	9.47E-07
PGS:BMI:BMI_imp	-1.56E-04	0.000229	0.495766
PGS:BMI:wb_fat_perc	0.002234	0.004331	0.605927
PGS:BMI_imp:wb_fat_perc	-0.00172	0.004329	0.69185
BMI:BMI_imp:wb_fat_perc	-8.66E-04	5.11E-06	0
PGS:BMI:BMI_imp:wb_fat_perc	-5.68E-06	5.03E-06	0.258385

Supplementary Table 22. Interactions Waist to hip ratio ~ BD PGS_{oxt}

Term	Estimate	Error	P value
(Intercept)	0.714302	0.005439	0
PGS	4.89307	0.552178	7.93E-19
grip_strength	-0.00139	8.48E-05	2.72E-60
genetic_sexmale	0.128835	2.90E-04	0
age	0.003915	1.72E-04	4.88E-115
age_sq	-2.35E-05	1.55E-06	4.31E-52
PC1	-0.20768	0.094625	0.02818
PC2	-0.03025	0.067003	0.651701
PC3	0.472682	0.067135	1.91E-12
PC4	-1.46049	0.068685	2.74E-100
PC5	0.291004	0.067175	1.48E-05
PC6	0.032297	0.066998	0.629767
PC7	-0.1599	0.067013	0.017026
PC8	-0.3017	0.067065	6.84E-06
PC9	0.030006	0.066992	0.654222
PC10	-0.85584	0.067375	5.80E-37
PGS:grip_strength	-0.13687	0.01688	5.14E-16

Supplementary Table 23. Interactions Waist to hip ratio ~ BD PGS_{oxt} PC

Term	Estimate	Error	P value
(Intercept)	0.689939	0.0047	0
PGS	0.002039	2.84E-04	6.63E-13
grip_strength	-7.10E-04	1.33E-05	0
genetic_sexmale	0.12884	2.90E-04	0
age	0.003917	1.72E-04	3.47E-115
age_sq	-2.36E-05	1.55E-06	3.40E-52
PC1	-0.20796	0.094627	0.027972
PC2	-0.03044	0.067002	0.649584
PC3	0.474191	0.067081	1.56E-12
PC4	-1.46723	0.068163	1.00E-102
PC5	0.292313	0.067116	1.33E-05
PC6	0.032659	0.067	0.625935
PC7	-0.16028	0.067012	0.016767
PC8	-0.30236	0.067056	6.51E-06
PC9	0.029643	0.066992	0.658138
PC10	-0.86152	0.06737	1.94E-37
PGS:grip_strength	-5.29E-05	8.68E-06	1.09E-09

Supplementary Table 24. Interactions Grip strength ~ BD PGS$_{oxt}$ PC2

Term	Estimate	Error	P value
(Intercept)	37.04693	0.513714	0
PGS	-0.38992	0.100783	1.09E-04
waist_hip_ratio	-8.2199	0.154155	0
genetic_sexmale	17.16392	0.027511	0
age	0.034871	0.018469	0.059022
age_sq	-0.00277	1.67E-04	3.65E-62
PC1	-8.13517	10.17502	0.423987
PC2	25.06201	7.204175	5.04E-04
PC3	4.523343	7.210752	0.530459
PC4	190.0853	7.29843	1.96E-149
PC5	-12.0052	7.213514	0.096059
PC6	-41.0647	7.203964	1.20E-08
PC7	77.71676	7.204634	3.99E-27
PC8	139.759	7.206879	9.62E-84
PC9	1.177852	7.20302	0.870108
PC10	389.0111	7.223936	0
PGS:waist_hip_ratio	0.483966	0.114967	2.56E-05

Supplementary Table 25. Interactions Waist to hip ratio ~ T2D PGS_{oxt} PC

Term	Estimate	Error	P value
(Intercept)	0.690224	0.004699	0
PGS	0.003721	2.86E-04	8.79E-39
grip_strength	-7.09E-04	1.33E-05	0
genetic_sexmale	0.128814	2.89E-04	0
age	0.003905	1.72E-04	1.67E-114
age_sq	-2.35E-05	1.55E-06	1.07E-51
PC1	-0.20464	0.094616	0.030554
PC2	-0.02319	0.067006	0.729279
PC3	0.450664	0.067219	2.02E-11
PC4	-1.37729	0.069695	6.91E-87
PC5	0.26601	0.06726	7.66E-05
PC6	0.031115	0.066991	0.642318
PC7	-0.15513	0.067005	0.020603
PC8	-0.29456	0.067054	1.12E-05
PC9	0.031296	0.066985	0.640348
PC10	-0.80111	0.067564	2.00E-32
PGS:grip_strength	-9.82E-05	8.72E-06	2.07E-29

Supplementary Table 26. Interactions Grip strength ~ T2D PGS_{oxt}

Term	Estimate	Error	P value
(Intercept)	37.04054	0.513719	0
PGS	0.391681	0.101202	1.09E-04
waist_hip_ratio	-8.21387	0.154156	0
genetic_sexmale	17.16324	0.027511	0
age	0.034954	0.01847	0.058423
age_sq	-0.00278	1.67E-04	3.37E-62
PC1	-7.99669	10.17501	0.431918
PC2	24.9194	7.204512	5.43E-04
PC3	4.666415	7.213406	0.517691
PC4	189.5	7.327939	2.37E-147
PC5	-11.7381	7.216936	0.10385
PC6	-40.9791	7.204003	1.28E-08
PC7	77.76515	7.204797	3.72E-27
PC8	139.9373	7.207567	6.17E-84
PC9	1.075109	7.203412	0.881356
PC10	389.1402	7.223368	0
PGS:waist_hip_ratio	-0.48152	0.11541	3.02E-05

Supplementary Table 27. Interactions Grip strength ~ T2D PC2 PGS_{oxt}

Term	Estimate	Error	P value
(Intercept)	37.04222	0.513678	0
PGS	0.760555	0.102219	1.01E-13
waist_hip_ratio	-8.21594	0.154155	0
genetic_sexmale	17.16175	0.02751	0
age	0.035061	0.018468	0.057636
age_sq	-0.00278	1.67E-04	2.85E-62
PC1	-8.38591	10.17434	0.409814
PC2	24.31364	7.205213	7.40E-04
PC3	7.107353	7.22855	0.325493
PC4	181.6669	7.492906	8.89E-130
PC5	-9.56258	7.23274	0.186127
PC6	-40.8531	7.20349	1.42E-08
PC7	77.65397	7.204147	4.35E-27
PC8	139.9775	7.206925	5.35E-84
PC9	0.880336	7.203055	0.902727
PC10	386.2701	7.237755	0
PGS:waist_hip_ratio	-0.94066	0.116566	7.06E-16

Supplementary Table 28. Interactions Energy intake ~ T2D PC PGS_{oxt}

Term	Estimate	Error	P value
(Intercept)	4232.209	198.3322	6.35E-101
PGS	-207.011	25.01565	1.29E-16
mean_food_intake	0.880818	0.008122	0
mean_sugar_intake	28.20377	0.180519	0
genetic_sexmale	932.5566	8.118862	0
age	-58.6169	7.257148	6.67E-16
age_sq	0.384052	0.065916	5.67E-09
PC1	5519.734	3939.157	0.161142
PC2	7004.507	2803.669	0.012479
PC3	-16259.6	2841.591	1.05E-08
PC4	49771.5	3178.036	3.00E-55
PC5	-11554.3	2837.954	4.68E-05
PC6	-694.044	2791.385	0.803641
PC7	-1377.52	2808.344	0.623774
PC8	1178.437	2843.4	0.678548
PC9	4878.304	2792.983	0.080703
PC10	14416.37	3007.604	1.64E-06
PGS:mean_food_intake	0.062762	0.007712	4.04E-16
PGS:mean_sugar_intake	1.710715	0.169659	6.64E-24
mean_food_intake:mean_sugar_intake	4.53E-05	4.23E-05	0.283853
PGS:mean_food_intake:mean_sugar_intake	-4.75E-04	3.77E-05	2.24E-36

Supplementary Table 29. Interactions Ability to confide ~ T2D PGS_{oxt}

Term	Estimate	Error	P value
(Intercept)	-2.71082	0.186503	7.27E-48
PGS	0.010728	0.007851	0.171812
loneliness1	1.082408	0.014791	0
family_friends1	0.06828	0.053391	0.200942
social_activity1	-0.01623	0.088992	0.855304
social_activity0	-0.20998	0.009488	1.59E-108
genetic_sexmale	0.585442	0.007596	0
age	0.015418	0.006797	0.023303
age_sq	2.89E-05	6.11E-05	0.635831
PC1	-3.68219	3.697251	0.319285
PC2	-1.03507	2.610839	0.691771
PC3	16.31783	2.607251	3.88E-10
PC4	-59.4773	2.514838	1.17E-123
PC5	18.13618	2.601359	3.13E-12
PC6	1.566825	2.620631	0.54992
PC7	4.61343	2.620795	0.078354
PC8	2.597545	2.61298	0.320178
PC9	-4.10624	2.617853	0.116752
PC10	-16.4466	2.633837	4.26E-10
PGS:loneliness1	-0.03495	0.014732	0.017688
PGS:family_friends1	-0.00609	0.053466	0.909386
loneliness1:family_friends1	-0.48166	0.08279	5.96E-09
PGS:social_activity1	-0.03443	0.084045	0.68207
PGS:social_activity0	-0.00279	0.009494	0.768507
loneliness1:social_activity1	-0.25096	0.15892	0.114305
loneliness1:social_activity0	-0.08254	0.018247	6.09E-06
family_friends1:social_activity1	-0.23556	0.574129	0.681596
family_friends1:social_activity0	-0.09266	0.063187	0.142538
PGS:loneliness1:family_friends1	-0.07974	0.084894	0.347572
PGS:loneliness1:social_activity1	-0.30027	0.164151	0.067367

PGS:loneliness1:social_activity0	0.006272	0.018232	0.730859
PGS:family_friends1:social_activity1	-0.10077	0.558306	0.85677
PGS:family_friends1:social_activity0	-0.00337	0.062946	0.957288
loneliness1:family_friends1:social_activity1	1.374151	1.011204	0.17417
loneliness1:family_friends1:social_activity0	0.025474	0.100473	0.799849
PGS:loneliness1:family_friends1:social_activity1	-1.66416	1.199331	0.165267
PGS:loneliness1:family_friends1:social_activity0	0.094256	0.102266	0.356697
